# The Mechanism of Toxicity in HET-S/HET-s Prion Incompatibility

**DOI:** 10.1371/journal.pbio.1001451

**Published:** 2012-12-27

**Authors:** Carolin Seuring, Jason Greenwald, Christian Wasmer, Roger Wepf, Sven J. Saupe, Beat H. Meier, Roland Riek

**Affiliations:** 1 Laboratory of Physical Chemistry, ETH Zürich, Zürich, Switzerland; 2 Electron Microscopy ETH Zurich (EMEZ), Zürich, Switzerland; 3 Laboratoire de Génétique Moléculaire des Champignons, Institut de Biochimie et Génétique Cellulaires, UMR-5095 CNRS/Université de Bordeaux 2, Bordeaux, France; Whitehead Institute, United States of America

## Abstract

The HET-s protein from the filamentous fungus *Podospora anserina* is a prion involved in a cell death reaction termed heterokaryon incompatibility. This reaction is observed at the point of contact between two genetically distinct strains when one harbors a HET-s prion (in the form of amyloid aggregates) and the other expresses a soluble HET-S protein (96% identical to HET-s). How the HET-s prion interaction with HET-S brings about cell death remains unknown; however, it was recently shown that this interaction leads to a relocalization of HET-S from the cytoplasm to the cell periphery and that this change is associated with cell death. Here, we present detailed insights into this mechanism in which a non-toxic HET-s prion converts a soluble HET-S protein into an integral membrane protein that destabilizes membranes. We observed liposomal membrane defects of approximately 10 up to 60 nm in size in transmission electron microscopy images of freeze-fractured proteoliposomes that were formed in mixtures of HET-S and HET-s amyloids. In liposome leakage assays, HET-S has an innate ability to associate with and disrupt lipid membranes and that this activity is greatly enhanced when HET-S is exposed to HET-s amyloids. Solid-state nuclear magnetic resonance (NMR) analyses revealed that HET-s induces the prion-forming domain of HET-S to adopt the β-solenoid fold (previously observed in HET-s) and this change disrupts the globular HeLo domain. These data indicate that upon interaction with a HET-s prion, the HET-S HeLo domain partially unfolds, thereby exposing a previously buried ∼34-residue N-terminal transmembrane segment. The liberation of this segment targets HET-S to the membrane where it further oligomerizes, leading to a loss of membrane integrity. HET-S thus appears to display features that are reminiscent of pore-forming toxins.

## Abstract 2

A nontoxic functional prion activates toxicity in the HET-S/HET-s fungal heterokaryon incompatibility system by converting HET-S into a cytotoxic membrane protein.

## Author Summary

Filamentous fungi have the potential for genetically distinct individuals to fuse, resulting in a cell with multiple nuclei known as a heterokaryon. This fusion event is controlled by genetic variants that determine the compatibility of the individuals, such that the fusion of incompatible genotypes triggers a cell death reaction in the heterokaryon. We have investigated the molecular mechanism of toxicity in the HET-S/HET-s incompatibility system in the fungus *P. anserina*. HET-s is an infectious yet non-toxic protein (prion) whose interaction with the almost identical protein HET-S has been shown to re-localize HET-S to the cell periphery, an event that is associated with the death of heterokaryons that simultaneously contain both proteins. We find that the HET-s prion converts soluble HET-S into a protein that binds to and destabilizes lipid membranes. Furthermore, we identify a potential transmembrane helix that is normally buried within the soluble fold of HET-S and show that its presence is associated with toxicity. We conclude that upon interaction with a HET-s prion, the HET-S globular domain partially unfolds, exposing a previously buried transmembrane segment that targets HET-S to the membrane. Once there, it further oligomerizes into a structure that causes a loss of membrane integrity, reminiscent of the mode of action of pore-forming toxins.

## Introduction

Amyloids have long been associated with dozens of diseases including Alzheimer, Parkinson, and prion diseases [1]. However, there are also amyloids with normal biological activities termed “functional amyloids” [2] of which the HET-s prion of the filamentous fungus *P. anserina* is an interesting example. This prion protein controls an allorecognition process known as heterokaryon incompatibility. Filamentous fungi have developed self/non-self recognition systems that prevent the vegetative fusion of individuals that differ in specific loci termed *het*-loci [3],[4]. The fusion of incompatible strains results in the rapid destruction of the fusion cell or heterokaryon in a process known as heterokaryon incompatibility. This limitation of cytoplasmic exchanges between unlike strains could act as a protection against the transmission of parasites [5] such as mycoviruses, senescence plasmids, or parasitic nuclei [6]. The number of *het-*incompatibility loci varies from species to species and although a number of *het*-loci have been characterized over the years in *P. anserina*, *Neurospora crassa*, and *Cryphonectria parasitica*, the mechanistic modalities of cell death remain largely elusive [6],[7]. The *het-s* locus is one of nine *het*-loci in *P. anserina* and the only one involving a prion protein [8]. The *het-s* locus exists as two incompatible allelic variants termed *het-s* and *het-S*. A strain that expresses the HET-s prion in its soluble form has the [Het-s*] phenotype (phenotypes are denoted throughout with square brackets) and is compatible with *het-S* strains. However, the HET-s protein can spontaneously aggregate in vivo to form an infectious amyloid yielding the [Het-s] phenotype that is incompatible with [Het-S] strains (those that harbor the HET-S protein) [9]. Because HET-s is a prion, when a [Het-s*] strain fuses with a [Het-s] strain, the amyloid form spreads from one to the other and in the end they both display the [Het-s] compatibility phenotype.

The molecular details of the infectivity of the HET-s prion have been studied in detail. The HET-s and HET-S proteins have two domains: an N-terminal globular domain (HeLo) and a C-terminal prion-forming domain (PFD) [10]. HET-s can function in vivo without its HeLo domain, i.e., its PFD (fused to GFP) is able to carry out the functions of infectivity and incompatibility [10]–[12]. HET-S, although 96% identical to HET-s [13], cannot form a prion, is not infectious, and does not readily form amyloids in vitro, yet it still requires both its HeLo domain and an intact PFD for its function in incompatibility [10],[14]. The HeLo domain of HET-S has also been shown to inhibit in cis the in vivo prion formation of its own PFD [10] as well as in trans the in vivo propagation of [Het-s] [15] and the in vitro aggregation of the HET-s [14]. The 3D structures of the HeLo domains of HET-s and HET-S [14] as well as of the infectious amyloid form of HET-s [16]–[19] have shed some light on the molecular details of infectivity and have confirmed the domain boundaries first identified by protease protection of the soluble and amyloid forms of HET-s [10],[20]. In solution, the HeLo domain comprising residues 1–227 is a mostly helical domain followed by a C-terminal disordered PFD domain comprising residues 228–289. Upon aggregation into the infectious state, the PFD of HET-s adopts a highly ordered β-solenoid structure [17] that is the infectious entity of the HET-s prion [10],[11],[16],[19]. The ten amino acid residue overlap of the two domains (218–227) means that the HeLo domain must at least partially unfold during the β-solenoid formation of the PFD, a statement that is supported by solid-state nuclear magnetic resonance (NMR) data [19].

In contrast to the mechanism of infectivity, little is known about the mechanism of toxicity in the HET-S/HET-s heterokaryon incompatibility reaction. In vivo both the HET-s prion and HET-S are required to produce toxicity, whereas the co-presence of soluble HET-s and HET-S proteins does not affect fungal viability [9],[21]. A recent report demonstrated that a relocalization of HET-S to the membrane periphery is associated with toxicity and that the cytoplasmic co-aggregates of HET-S and HET-s are not toxic [12]. The authors also show that the mechanism of HET-s prion-mediated HET-S toxicity functions in yeast as well as in filamentous fungi. Herein, we present insights into the origin of toxicity of HET-S/HET-s mixtures in which the HeLo domain of HET-S is directly involved in effecting membrane damage. Our data indicate that HET-S binds to the HET-s amyloid through its PFD domain thereby forming a unit of the β-solenoid structure. The beta structuring of the HET-S PFD leads to a destabilization and partial unfolding of the HET-S HeLo domain upon which an N-terminal segment of ∼30 amino acid residues is expelled from the HeLo fold. This segment, which is predicted to be a transmembrane (TM) helix, is found to anchor the protein in the membrane and to convert HET-S into an integral membrane protein. In the lipid bilayer, HET-S oligomerizes into complexes that perforate the membrane thus leading to toxicity via membrane leakage.

## Results

### Prion-Induced Membrane Damage by the HET-S Protein Can Be Reconstituted In Vitro

**Figure 1:**
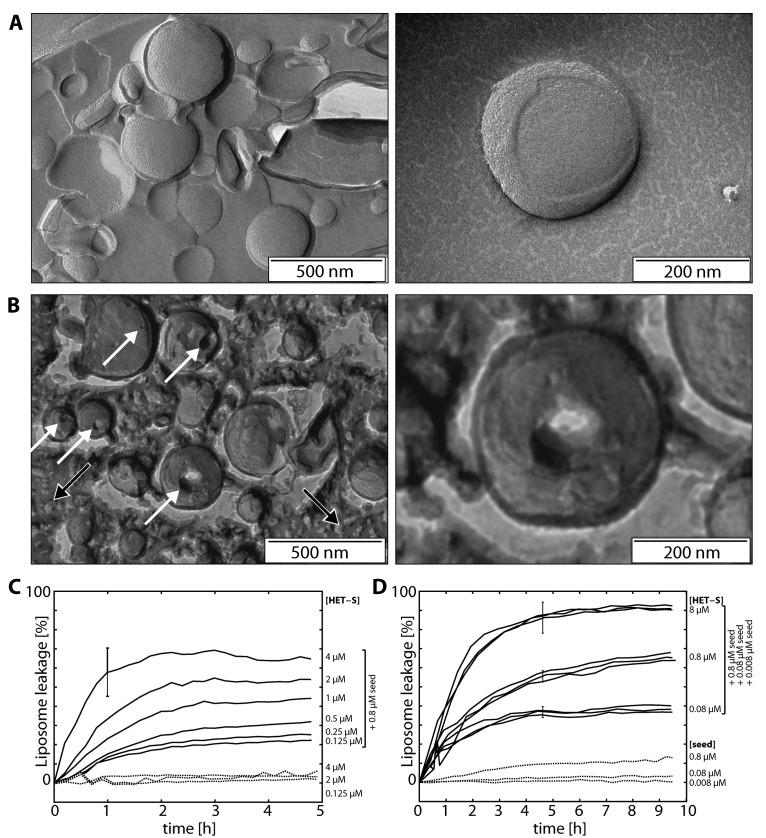
HET-S in the presence of HET-s(218–289) amyloid seeds makes holes in liposomes observed by freeze-fracture electron microscopy and liposome leakage assays. (A) TEM images of the replica of 100-nm diameter extruded liposomes incubated at 4°C in the absence of protein, with a higher magnification on the right. (B) Liposomes incubated in the presence of a mixture of HET-S and HET-s(218–289) fibril seeds display membrane damage: Hole-like structures ranging from a ∼10–∼60-nm width indicated by white arrows are present. In addition, species interpreted as protein aggregates are labeled by a black arrow. (C) The time-dependent release of calcein from *E. coli* polar liposomes induced by HET-S and HET-s(218–289) 10°C was measured at HET-S concentrations of 0.12–4 µM in the presence (solid lines) or absence (dashed lines) of 0.8 µM HET-s(218–289) amyloid seeds (monomer-equivalent concentration). (D) The calcein leakage measured in the presence (solid lines) or absence (dashed lines) of 8, 0.8, or 0.08 µM HET-S with either 0.008, 0.08, or 0.8 µM HET-s(218–289) amyloid seeds.

Recent results indicated that the HET-s prion triggers HET-S to relocalize at the cell membrane (in *Podospora* and yeast), and that this event is associated with toxicity [12]. In order to further probe the determinants of toxicity, we sought to reproduce the observed phenomenon in vitro with purified proteins and liposomes composed of *Escherichia coli*-derived polar lipids. Freshly extruded 100-nm diameter liposomes were incubated for 1 h at 4°C with HET-S with or without approximately equimolar amyloid seeds (the prion form) of the HET-s PFD, HET-s(218–289). We use the latter protein construct as it has been shown to be sufficient in vivo for HET-s infectivity and as a GFP-fusion sufficient for incompatibility [10],[11]. The liposome samples were then frozen in liquid ethane and visualized by freeze-fracture electron microscopy. Transmission electron microscopy (TEM) images of samples that had been incubated at 4°C in the absence of protein revealed liposomes with smooth surfaces (Figure 1A). In the combined presence of HET-S and HET-s(218–289) amyloid seeds, the liposomal membranes are severely disrupted, with more than half of the liposomes displaying defects of 10 to 60 nm in size (white arrows in Figure 1B). In addition, the space between liposomes in the protein-containing sample is filled with small aggregates (indicated by a black arrow in Figure 1B) similar in size to those documented in liposome-free co-aggregation assays of HET-S with HET-s(218–289) [14]. In another experiment, the extruded 100 nm liposomes were incubated for 1 h at 4°C with HET-S only or HET-S with approximately equimolar HET-s(218–289) amyloid seeds. The results (Figure S1) show that at 4°C only the combination of HET-S with amyloid seeds leads to defects in the liposome membrane.

The size of the defects found in the TEM images suggest that they should lead to a significant loss of liposome integrity, so we employed a dye leakage assay to observe the effects of HET-S on liposomal membranes under various conditions. Using liposomes filled with 60 mM calcein, a concentration at which significant self-quenching occurs, we could simultaneously measure kinetics for the efflux of liposomal contents for many samples with a fluorescence plate reader [22]. As the calcein leaves the interior of the liposome, it is diluted more than 10,000-fold leading to a fluorescence increase that is monitored at 520 nm with excitation at 485 nm. Triplicate measurements were made for samples with a range of protein concentration: 125 nM–8 µM HET-S and 125 nM–4 µM HET-s(218–289) seeds (monomer-equivalent concentration). We performed the experiments at 10°C in order to minimize the volume change due to evaporation during the 6-h-long measurements. Additionally, the liposomes were more stable at 10°C than at room temperature, leading to a lower background leakage. The results, plotted in Figure 1C and 1D, demonstrate that significant liposome leakage is only observed for the samples that contain both HET-S and HET-s(218–289) amyloid seeds. In the range of concentrations tested, the individual proteins did not give rise to significant leakage in the time course of the experiment at 10°C. Comparison of the kinetic curves in Figure 1C and 1D indicates that even though the amyloid seeds are required to get HET-S dependent leakage, the rate of leakage and maximum leakage is determined by the HET-S concentration and essentially independent of the seed concentration in the range tested. This finding indicates that the leakage induction requires an interaction between HET-S and a HET-s(218–289) prion but that very few seeds of HET-s(218–289) (at least 1,000× less than HET-S) are required to get the maximal effect.

### Prion-Independent Toxicity and Membrane Association of HET-S Observed in *E. coli*

**Figure 2:**
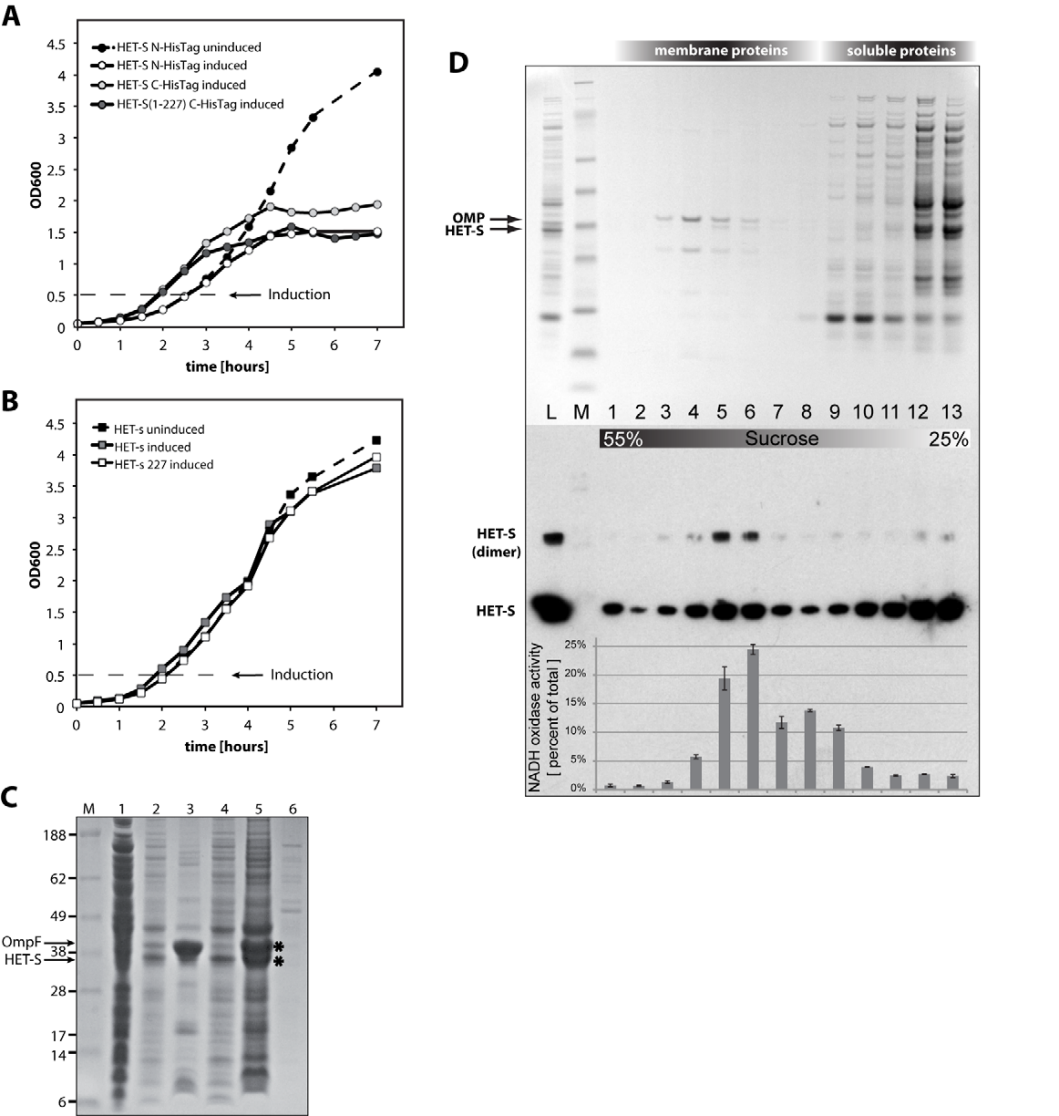
HET-S but not HET-s expression is toxic in *E. coli*. The growth of *E. coli* cultures expressing various constructs of (A) HET-S (full-length, 1–227, or N-terminal Histag) or (B) HET-s (full-length, 1–227) was monitored by their OD_600_. After induction of protein expression, the cell growth of cultures with HET-S or HET-S(1–227) but not HET-s or HET-s(1–227) was suppressed, indicating that the HET-S HeLo domain, but not HET-s or its HeLo domain, is toxic to *E. coli*. Further analysis of the cell pellet showed that HET-S is associated with the inner membrane of *E. coli*. (C) SDS-PAGE analysis of soluble and insoluble fractions after expression in *E. coli* at 37°C for 4 h. Lanes 1 and 2 represent the supernatant and pellet of the cell lysate, respectively. The pellet and supernatant fractions after treatment with 1 M urea are in lanes 5 and 6, respectively. HET-S remains in the insoluble fraction after incubation in 1 M Urea (lane 5).The pellet and supernatant fractions after extraction with the detergent N-lauroylsarcosine are shown in lanes 3 and 4, respectively, showing that HET-S is solubilized by this detergent. The identity of the HET-S and OmpF bands were confirmed by trypsin digest followed by MS analysis and are marked with an asterisk. (D) Coomassie stain (top) and Western blot (bottom) visualization of SDS-PAGE fractions from a sucrose density gradient separation of *E. coli* cell lysates after HET-S expression at 37°C for 4 h (lanes 1–13). Lanes L and M are the cell lysate before density gradient separation and the protein marker (SeeBlue Plus2, Invitrogen), respectively. The relative NADH oxidase activity of each fraction (indicating the location of the inner membrane) is plotted over the Western blot. HET-S is found in fractions that correspond to the inner membrane, whereas the outer membrane protein OmpF moves to a higher density. The majority of HET-S is in the soluble protein fractions while a small amount is also found on the very bottom of the tube (55% sucrose, fraction 1) whose density (>1.25 g/ml) indicates that it likely to be lipid-free (or low-lipid-content) protein aggregates.

We have long observed that upon induction of HET-S expression in *E. coli*, but not for HET-s induction, the host undergoes a growth arrest during expression at 37°C (Figure 2). Low temperature induction can partially overcome this toxicity, and we routinely use 18–25°C to minimize the amount of insoluble HET-S and maximize overall yield. In contrast, overexpressed HET-s is not toxic to *E. coli*. While HET-s is usually found in inclusion bodies (short inductions can yield some soluble protein), HET-S was always found to partition between a soluble and insoluble fraction, with longer induction times leading to a lower yield of soluble protein and more proteolytic degradation (unpublished data). In light of its membrane disruption activity (Figure 1), we wanted to investigate whether HET-S exerts its toxicity in *E. coli* by interacting with the host membrane. We found that upon induction at 37°C in *E. coli*, the insoluble fraction of HET-S was associated with the bacterial inner membrane. First, we observed that HET-S remains in the insoluble fraction when washing the membranes with 1 M urea but is solubilized by N-lauroylsarcosine. This detergent is known to not solubilize the outer membrane as exemplified by the observation that the outer membrane protein goes into the pellet (Figure 2C). We also fractionated *E. coli* cell lysates via isopycnic centrifugation using a 25%–55% (w/w) sucrose gradient, conditions known to resolve the two membranes of different densities [23]. SDS-PAGE analysis of the fractions revealed that the majority of HET-S was soluble while a significant fraction migrated with the membranes, having a density distribution that more closely matched the less dense inner membrane (visualized by NADH oxidase activity) than the outer membrane (visualized by OMP-F migration) (Figure 2D). In addition, there was a dense fraction that migrated to the bottom of the tube that, based on its high density, must either be lipid-free or low-lipid content aggregates. The identity of the HET-S and OmpF bands on the gel were confirmed by tryptic digest followed by MALDI-TOF analysis (unpublished data) and the migration of HET-S observed by western blot with an anti-His-tag antibody. Thus, in addition to its soluble form, HET-S occurs in a membrane-associated state and as insoluble protein aggregates when overexpressed in *E. coli*. The membrane association and toxicity of HET-S in *E. coli* suggests a mechanism of action that is similar to what has been seen in yeast and *Podospora* [12].

### Thermodynamic Control of HET-S Activation

**Figure 3:**
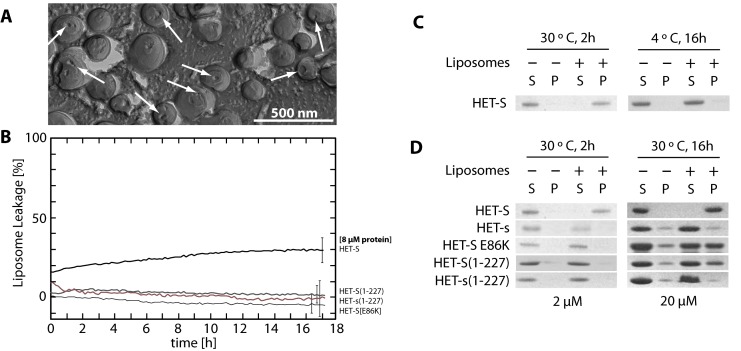
Prion-independent (thermodynamic) activation of HET-S reveals that membrane binding and liposome leakage are distinct activities and that liposome binding is mediated through the N-terminus of HET-S. (A) TEM image of freeze-fractured liposomes treated with HET-S at 30°C show that many liposomes contain a single large hole, indicated by white arrows similar to those with prion-activated HET-S (Figure 1B). Consistent with the TEM images, is (B) the prion-independent leakage caused by HET-S 30°C. The HET-s-like variant HET-S[E86K] and the HeLo domains of HET-S and HET-s do not cause leakage even at this elevated temperature. (C) SDS-PAGE analysis of 150,000 *g* supernatant (S) and pellet (P) fractions from a 20 µM HET-S incubation with liposomes reveals that HET-S associates with liposomes at 30°C but not at 4°C. (D) HET-s and HET-s(1–227) do not interact with liposomes and are found in the supernatant. In contrast, HET-S(1–227) and HET-S(E86K)—a HET-S mutant that cannot dimerize in solution and that has a [Het-s]-like phenotype in vivo—bind to liposomes after an extended period of incubation at 30°C. Despite being able to bind liposomes, neither of these two proteins causes leakage from liposomes (B).

In contrast to *Podospora*, where the HET-s prion is required for toxicity, HET-S is toxic in *E. coli* in the absence of a HET-s prion seed. Our previous report on the mechanism of prion inhibition by the HET-S HeLo domain found that a lower thermodynamic stability of the HeLo domain of HET-S compared to that of HET-s is associated with HET-S activity. Our data indicated that prion inhibition of HET-s by HET-S involves a complex with a destabilized HET-S HeLo domain, and we hypothesized that it may also be the precursor to the toxic entity [14]. Therefore we attempted to learn if at a temperature closer to the observed unfolding transition of the HET-S HeLo domain (48°C) prion-independent liposome disruption and leakage could be observed. The results depicted in Figure 3A show that at 30°C, HET-S can induce the same hole-like structures in liposomes as seen previously at 4°C in the presence of prion seeds, while the negative control with liposomes in absence of HET-S did not show such defects (unpublished data). Also, at 30°C HET-S can induce a low but significant level of liposome leakage without the HET-s prion (Figure 3B). Apparently, in vitro at elevated temperature HET-S is capable of some level of self-activation in the absence of HET-s seeds. Yet, this seed-independent liposome leakage activity of HET-S is not observed for the isolated HET-S HeLo domain (Figure 3B), further evidence of a role for the PFD region in HET-S activation. An analogous auto-activation of HET-S has not been observed even at elevated growth temperatures (37°C) in either *P. anserina* or yeast (unpublished data). It may be that the extent of auto-activation is too low to lead to a detectable in vivo toxicity or else that the chaperone machinery suppresses the toxic effect of this auto-activation.

### Liposome Binding Is Distinct from Liposome Leakage

Several reports have identified single amino-acid substitutions in HET-S that convert it to a protein that gives the [Het-s] phenotype in vivo [21],[24]. Such a high degree of structural and sequence similarity between two proteins that differ in their in vivo localization and activities is suggestive of a loss (or gain) of function mutation. In light of the membrane leakage data and EM images (Figures 1 and 3), the fact that all of the inter-converting mutations (HET-S↔HET-s) reside in the HeLo domain suggests that the HeLo domain of HET-S has a membrane disrupting function. In principle, this function should require a membrane-binding activity as well as a pore-forming activity. Since membrane binding may be more a permissive activity than a pore-forming activity, we set up an assay to measure liposome binding in order to see if the two activities are distinct. Liposomes were prepared as for the TEM analyses and proteins were incubated either at 4°C in the presence or absence of amyloid seeds (prion activation) or at 30°C without seeds (thermodynamic activation). The samples were then centrifuged at 150,000 *g*, and the pellets and supernatants analyzed by SDS-PAGE. The assay for the seeded activation of liposome binding suffers from the inability to separate the liposome-bound protein from simple prion-induced, lipid-independent aggregates; however, there is still significant and reproducible prion-activated binding of HET-S to the liposomes (unpublished data). The results for the thermodynamic activation are clearer and show that HET-S does but HET-s does not bind to liposomes (Figure 3C and 3D). We also tested the HeLo domain of HET-S alone using the construct HET-S(1–227), and it is able to associate with liposomes, whereas HET-s(1–227) is not. Finally we tested HET-S[E86K], a mutant that has lost its incompatibility with [Het-s], thus converting HET-S to a HET-s like protein [21]. However, unlike HET-s, HET-S[E86K] retains membrane binding activity. This means that membrane binding, as demonstrated by HET-S(1–227) and HET-S[E86K] is not sufficient for heterokaryon incompatibility. These same proteins were also subjected to the liposome leakage assay and only the full-length HET-S protein gives rise to leakage (Figure 3B). Thus, the [Het-S] phenotype of a protein is directly correlated with liposomal membrane leakage and not just with liposome binding.

### The HET-s Prion Triggers a Conformational Change in the HET-S HeLo Domain

**Figure 4:**
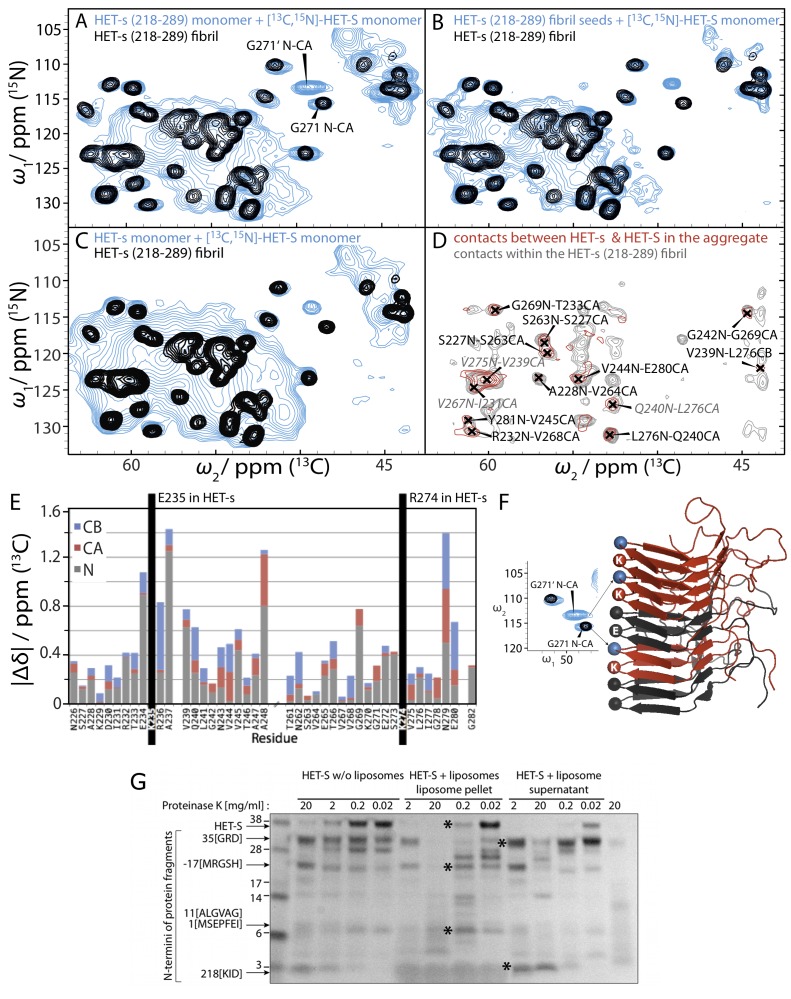
Solid-state NMR reveals that HET-S adopts the HET-s(218–289) β-solenoid fold upon aggregation induced by HET-s. NCA solid-state NMR spectra of various protein samples are shown. The NCA spectrum of the HET-s(218–289) fibrils alone is depicted in black contours in A, B, and C. The blue contours represent the NCA spectrum of [^13^C, ^15^N]-HET-S (A) co-fibrillized with unlabeled HET-s(218–289) monomer, (B) aggregated by amyloid seeds of unlabeled HET-s(218–289), (C) co-fibrillized with unlabeled monomeric full-length HET-s. For the amino acid residue G271, two cross-peaks are observed that are attributed to the presence of two interfaces in the mixed fibril sample, i.e., HET-S/HET-S and HET-S/HET-s. (D) Overlay of the PAIN [27] spectrum (in red) of aggregates formed by [^13^C]-HET-S and [^15^N]-HET-s(218–289) and the PAIN of HET-s(218–289). The PAIN spectrum shows cross peaks only if there is an intermolecular polarization transfer between the two differently labeled molecules. The peak assignments are based on the chemical-shift assignment obtained by comparison with HET-s(218–289). (E) Chemical-shift differences between the β-solenoid fold of HET-s(218–289) fibrils and the PFD of HET-S upon aggregation with HET-s(218–289) fibril seeds. The absolute values of the differences in the N, C^α^, and C^β^ chemical shifts between HET-s and HET-S for the residues assigned in HET-S (within the HET-s(218–289)/HET-S-aggregate) are displayed. Most chemical shift differences are within the errors of the measurements, which are about 0.2 ppm for ^15^N and 0.1 ppm for ^13^C. Larger shift differences are observed close to the amino acid difference between HET-S and HET-s, K235E. (**F**) A model of the PFD in the mixed aggregates depicting the interfaces that give rise to the two resonances for residue G271. The spectra show the relevant region from (**A**). The ratio of intensities G271∶G271′ indicates that there are an equal number of HET-S/HET-s and HET-S/HET-S interfaces in the aggregates from the 1∶1 mixture. (**G**) The N-terminus of HET-S remains associated to liposomes upon proteolytic degradation. Proteinase K-treatment (0°C, 30 min) of HET-S that had been pre-incubated in presence or absence of liposomes at 37°C was analyzed by SDS-PAGE. The liposome sample was centrifuged at 180,000 *g* for 30 min to separate the liposome-bound protein from the soluble protein. This analysis reveals that residues ∼1–35 of HET-S are associated with the liposome fraction and are protected from proteolysis by the non-specific protease PK. The bands indicated by an asterisk have been analyzed by Edman degradation. The N-terminal sequences of three major degradation products of the pelleted liposome-embedded material (masses of approximately 20 kDa and two at 7 kDa) are ^−17^[MRGSHHH] and ^1^[MSEPFEI] and ^11^[ALGVAG] as indicated. More bands could not be analyzed attributed to the high degree of overlap and low of concentration of the remaining bands. The two strongest bands in the supernatant of the protease-treated HET-S/liposome sample with approximate masses 28 kDa and 5 kDa and have N-terminal residues starting with ^35^[GRD] and ^218^[KID]. These two bands are consistent with fragments spanning residues 35–289 and 218–289 (the PFD) respectively. The first lane is the SeeBlue Plus2 protein standard and the last lane is PK alone. These data were reproduced three times.

A protein that can exist in both a soluble and a membrane-associated form is likely to undergo a structural change when moving from the more polar to the more hydrophobic environment. Faced with the finding that both thermal destabilization and the interaction with a prion can induce HET-S to adopt a membrane-associated form, we wanted to investigate the structure of the HET-S HeLo domain when it is in complex with the HET-s prion. Therefore co-aggregates of [^13^C, ^15^N]-labeled HET-S with non-labeled (and therefore NMR silent) HET-s or HET-s(218–289), formed by mixing them at a 1∶1 molar ratio as well as co-aggregates obtained by seeding with non-labelled HET-s(218–289) amyloid seeds, were investigated by solid-state NMR. Inspection of the NCA 2D spectra (Figure 4A–4C) immediately indicated that the spectra of HET-S contain the previously assigned narrow resonances of HET-s(218–289) [18],[25]. Upon sequential assignment of the spectra using the 3D correlation experiments NCACB, NCOCX, and CANCO [26], a good correlation between the chemical shifts of previously reported HET-s(218–289) aggregates and HET-S in the various co-aggregate samples was observed (Figure 4A–4C), indicating a very similar backbone and sidechain conformation. Only for residues near K235 (which is E235 in HET-s) and for residues A237, A248, and N279 were considerable chemical-shift differences observed (Figure 4G), however barely larger than one ppm, which is typically regarded as a value for significant structural differences. Thus, it is concluded that the PFD of HET-S assumes the HET-s β-solenoid fold [17] when it is co-aggregated with HET-s(218–289) or HET-s.

Since the conformational change of the PFD of HET-S appears to be triggered by the presence of HET-s(218–289) it is likely that the two proteins interact with each other in the β-solenoid structure in the same way as do the individual molecules within HET-s(218–289) amyloid. In order to confirm this type of inter-molecular interaction in the HET-S/HET-s(218–289) co-aggregates, an aggregated sample from ^13^C-labeled HET-S and ^15^N-labeled HET-s(218–289) was prepared. The presence of cross peaks in a ^13^C-^15^N polarization transfer experiment (in the present case PAIN) [27],[28] is a direct proof of molecular-level contacts between HET-S and HET-s(218–289), thus indicative of an intimate mixing of the two proteins in the fibrils. The PAIN spectrum of the mixed sample of ^13^C-labelled HET-S and ^15^N-labelled Het-s(218–289) given in Figure 4D, allowed us to assign a number of hetero-intermolecular backbone-backbone contacts. Almost all peaks are explained by in-register contacts (residue peaks (i→i+36) between HET-S and HET-s(218–289) [17], establishing that the intermolecular interface between the residues involved in the β-solenoid is the same between HET-S and HET-s(218–289) as between two HET-s(218–289) monomers in pure fibrils. All the peaks observed are also present in the mixed sample of HET-s(218–289) (Figure 4D, grey contours), although the signal-to-noise in the HET-S/HET-s(218–289) spectrum is smaller due to the larger molecular mass of HET-S and hence fewer molecules in the sample. Further support for the hetero-intermolecular interaction within the β-solenoid is the observation that G271 of HET-S shows a resonance doubling. This indication of a structural heterogeneity is expected for a mixed amyloid of HET-S and HET-s(218–289) because in the β-solenoid structure, G271 is adjacent to residue 235 in the neighboring molecule (G235 in HET-s and K235 in HET-S, depicted in Figure 4F). Comparison of the peak-intensities for G271 and G271′ in the 1∶1 HET-S:HET-s(218–289) co-aggregates indicates that the HET-S/HET-s and HET-S/HET-S interfaces are roughly equally abundant, indicating a random mixing of the two monomers.

While the PFD segment of HET-S in these coaggregates with HET-s(218–289) displays narrow NMR signals attributed to the formation of the β-solenoid structure, the HeLo domain (residues 1–220) has very broad peaks (Figures 4A–4C and S2). An analogous observation has been documented for the HeLo domain in full-length HET-s fibrils [19] and points to a loss of the well-defined tertiary structure in the globular HeLo domain of HET-s induced by the β-solenoid fibril formation of its PFD. The similarity of the spectra obtained for HET-S and HET-s aggregates indicates that both HeLo domains undergo a similar structural rearrangement. Since the HeLo domain in its soluble form and the PFD in its amyloid form both include residues 218–227, it is rationalized that this domain overlap induces a global loss of tertiary structure in the HeLo domain upon amyloid formation of the PFD. This is consistent with the finding that decoupling the HeLo domain from the PFD β-solenoid conformation, by a construct that fuses the HET-S globular domain (1–227) to the PFD region (218–289) with a six amino-acid linker between the two domains, results in a loss of the HET-S heterokaryon incompatibility activity [14].

### HET-S Changes Its Conformation upon Lipid-Association

To probe the structural differences between the soluble versus lipid-associated forms of HET-S we chose limited proteolysis with proteinase K (PK) since it has already proven useful for the characterization of the domain boundaries of the soluble and amyloid forms of HET-s [20]. For this experiment, HET-S was pre-incubated for 1 h with and without liposomes at 37°C followed by proteolysis with varying concentrations of PK. The liposome sample was centrifuged at 150,000 *g* for 20 min to collect the digested protein fragments that remain associated with liposomes and this fraction was analyzed side by side with the liposome-free sample by SDS-PAGE. Significant differences in the protection pattern as well as with the overall sensitivity of HET-S to proteolysis were observed. The results in Figure 4G show that HET-S becomes more sensitive to PK digestion when bound to liposomes. Several of the digested bands could be analyzed successfully by N-terminal Edman degradation, giving insights into the parts of the protein that remain associated to the lipids after proteolysis. Of particular interest is that the fragments that remained associated with the liposome and that could be unambiguously identified (the most abundant) all contain at least 24 of the first 34 residues (Figure 4G), while a major band in both the non-liposomal HET-S digest and in the supernatant of the liposome digest has residue 35 as its N-terminus. In summary, the limited proteolysis data indicate that the liposome association occurs via an N-terminal segment and that it induces an increase in protease sensitivity. An increased proteolytic sensitivity indicates a loss of tertiary structure, consistent with what was observed in the NMR experiments when HET-S was exposed to HET-s(218–289) prion seeds (see above).

### Transmembrane Helix Prediction Correlates with Liposome Leakage and Phenotype

During routine bioinformatics analyses of HET-S and HET-s we discovered a “hidden” TM helix in the HeLo domain. Several algorithms predict with varying degrees of certainty that an N-terminal stretch of residues in the HeLo domain is a TM helix. HMMTOP [29] predicts one in both HET-S (residues 7–25) and HET-s (residues 5–22). TOPPRED [30] also predicts a TM helix but with more certainty for HET-S (residues 5–25) than HET-s (residues 2–22), the latter scored as “putative.” TMpred, which is based on the TMbase [31] database of membrane proteins, also predicted similar regions to be TM helices with a higher score for HET-S (residues 7–25) than HET-s (residues 4–22). While it is clear that there is some TM character to the HeLo domain, another prediction algorithm, TMHMM [32], appears more discerning. The output of the TMHMM algorithm includes a per residue probability that is used to calculate the overall TM score for a stretch of amino acids. While the prediction algorithm is more complex than these simple scores imply, TM helices in membrane proteins generally have per residue probabilities above 0.7 and total scores (the sum of the per residue probabilities within a single stretch) of at least 17. While TMHMM predicts neither HET-S nor HET-s to be membrane proteins, there is a considerable signal in the HET-S sequence (TM score = 9) that is absent in the HET-s sequence (Tm score = 0.5). Therefore we used TMHMM for all further predictions of the HET-S/s variants that interconvert the protein activities. We also found that shorter sequences gave higher scores, so that when residues 1–45 instead of 1–289 are submitted to the TMHMM-2.0 server, HET-S is predicted to have a TM helix (TM score = 18) while HET-s is still not (TM score = 4). Experimental support for these TM predictions is that the N-terminus of HET-S remains bound to HET-S/liposome mixtures treated with PK (Figure 4G).

**Figure 5:**
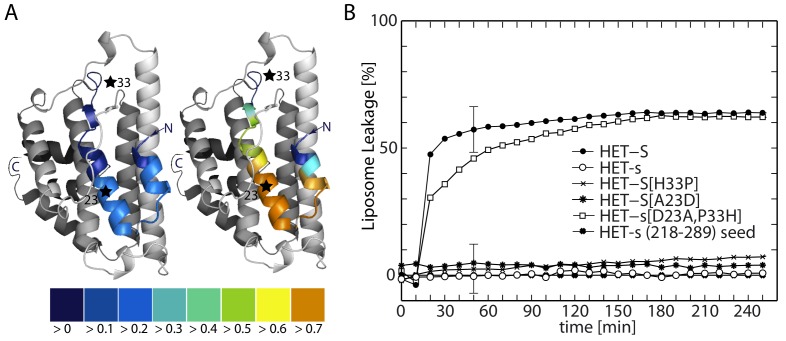
Correlations between *P. anserina* heterokaryon incompatibility phenotype and in vitro liposome calcein leakage data. (A) The TMHMM per-residue predictions (see also Figure S3) for HET-s (left) and HET-S (right) are mapped as a color gradient onto a ribbon diagram of the crystal structure of HET-S(1–227). A star indicates the locations of the two critical amino acid residues, 23 and 33, whose identities can interconvert the functionality of HET-s and HET-S. (B) The time course of prion-induced calcein leakage is shown for HET-s, HET-S, and several phenotype-interconverting variants at 4 µM protein concentration with 0.8 µM HET-s(218–289) amyloid seeds. Only HET-S and HET-s[D23A,P33H] show calcein leakage consistent with the HET-S toxic phenotype. The leakage is expressed as fraction based on the positive control experiment using 1% SDS. A comparison of the TM prediction, incompatibility phenotype, and liposome leakage activity of these variants is presented in Table S1. A detailed per-residue TMHMM prediction of these and several other variants of known phenotype is displayed in Figure S3).

To further test the relationship between the TM prediction, the in vitro observations of membrane leakage and the in vivo incompatibility phenotype, we initiated a study of some of the well-known variants of HET-s and HET-S. Of the 13 amino acid differences in the sequences HET-s and HET-S it is sufficient to replace a single residue in HET-S (H33P) to convert the protein to a HET-s-like protein that has the [Het-s] compatibility phenotype in *P. anserina*. Conversely, minimally two substitutions are required in HET-s (D23A and P33H) in order to switch the phenotype from [Het-s] to [Het-S] [24]. In other words, in vivo HET-s[D23A, P33H] leads to toxicity upon confrontation with a strain that carries the HET-s prion, while a *P. anserina* host that carries the HET-S[H33P] variant is compatible upon fusion with a strain bearing a HET-s prion. In fact these HET-S variants confer all the qualities of [Het-s] including infectivity, prion formation, and incompatibility with HET-S bearing strains. The results of the liposome leakage assay with these variants are depicted in Figure 5. There is a near perfect correlation between the TMHMM predictions, the leakage data, and the observed phenotype. The only exception to the correlation is the positive TM prediction for HET-S(H33P), as this variant actually gives a [Het-s] phenotype and does not cause liposome leakage. Thus, while the TM prediction is a good indicator of phenotype, the correlation between liposome leakage and HET-S-like toxicity is 100% (Figure 5; Table S1).

### HET-S Oligomer Formation in the Presence of a Membrane-Like Environment

**Figure 6:**
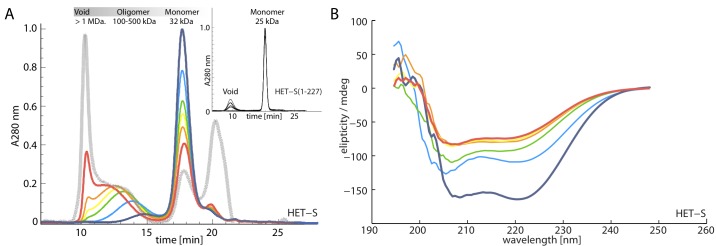
HET-S oligomerizes in the membrane-like environment of the detergent FC-12. (A) The time dependence of the oligomerization state of HET-S in 0.4% FC-12 was followed by size-exclusion chromatography with multiple-angle light scattering, UV, and refraction index detection. With time the monomeric species is depleted with a concomitant increase in the oligomeric one (violet = 0 h; blue = 1 h; green = 2.5 h; yellow = 3 h; orange = 3.5 h; red = 16 h). The grey trace is the size exclusion profile of HET-S that was extracted by 0.4% FC-12 from liposomes with which it had been incubated at 37°C for 2 h. The extracted sample is similar in monomer and oligomer content to the 16-h non-liposome measurement. The masses of the HET-S entities (not including the bound FC-12 molecules) as calculated by the Astra V software conjugate analysis are listed on above the chromatograms. The inlay shows that under the same conditions, HET-S 1–227 remains monomeric for 16 h with only a minor accumulation of a high molecular weight aggregate (no intermediate oligomers detected). (B) CD spectra of HET-S in 0.4% FC-12 were independently acquired for the same time course showing a time-dependent loss of alpha-helical content for the first 3 h upon the addition of detergent, after which the sample reaches a stable alpha helical content. Colors are as in (A).

If, as our data suggest, HET-S exerts a toxic activity by damaging the fungal membrane through the formation of an integral membrane protein, then this raises the question of how a membrane protein with a single TM helix is able to induce liposome leakage and the large observed defects in the liposomal membrane (Figure 1). In light of the observation that HET-S(1–227) is able to bind to liposomes without causing liposome leakage, a simple explanation of the mechanism of toxicity is that HET-s amyloid seeds not only trigger HET-S to form a membrane active complex, but also support its oligomerization into an aggregate species that then is both membrane active and toxic. Oligomerization-based mechanisms of membrane disruption are in fact common for many pore-forming toxins. Consistent with this type of mechanism is the finding that the HET-s-like variant HET-S[E86K], which has lost its ability to dimerize in solution, can still bind to the liposomes like HET-S(1–227) without causing liposome leakage however. In order to find support for the suggested oligomer-based mechanism we attempted to monitor the homo-oligomerization of HET-S in a membrane-like environment by size-exclusion chromatography with multi-angle light scattering (MALS), refraction index (RI), and ultraviolet (UV) detection. This triple detection scheme with MALS measurements allows for a model-free molecular weight determination of the protein and detergent components in a protein-detergent complex (PDC) [33]. For these measurements, recombinant HET-S was incubated at room temperature in 0.4% foscholine-12 (FC-12) or extracted by 0.4% FC-12 from liposomes with which it had been pre-incubated at 37°C (conditions shown to cause HET-S membrane binding and damage, Figure 6). When starting from soluble protein, the addition of FC-12 led to a time-dependent evolution of HET-S from a monomeric species to an oligomeric species of intermediate size and finally to a larger aggregate (Figure 6A). HET-S extracted from liposomes had a similar distribution of species as the 16-h incubation of soluble HET-S (Figure 6A). The mass of the protein component in the intermediate species was in the range of 100–500 kDa while the larger aggregates were >1 MDa (a precise measurement was not possible because the larger aggregates eluted in the void volume of the column and we therefore only measure a weight-average mass of any co-eluting species). Similar time-dependent oligomerization was observed with another detergent, n-Dodecyl-β-D-maltoside (unpublished data). To test the role of the PFD in this oligomer formation, HET-S(1–227) was treated with the same detergents. The HeLo domain alone remains monomeric for a longer time and does not form the intermediate-sized oligomers, rather going directly into >1 MDa aggregates (Figure 6A). We followed the same time-dependent processes by circular dichroism (CD) spectroscopy and found that HET-S continuously loses alpha-helical content on the same time scale as the aggregation, reaching a stable point at about 50% of the original signal (Figure 6B). This loss of structure is consistent with the solid-state NMR measurements of the HET-S aggregates (Figures 4 and S2) and the increase in PK sensitivity (Figure 4G).

## Discussion

### A Model for Prion-Induced Toxicity

**Figure 7:**
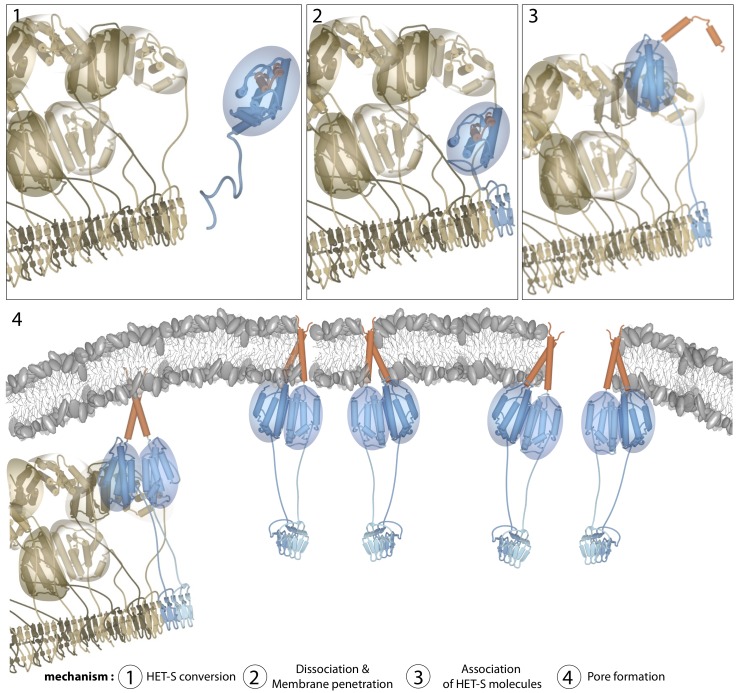
Proposed mechanism for the generation of toxicity by the HET-s prion/HET-S system. (1) In the fusion cell, HET-S (in blue with a red TM segment) encounters the β-solenoid structure of the HET-s prion (in brown). (2) HET-S binds to the β-solenoid structure through its own PFD segment, itself adopting the β-solenoid structure. The structural overlap of the HeLo domain and the PFD causes a partial unfolding of the HeLo domain of HET-S, represented here by the transition to a random coil conformation of its three C-terminal helices. (3) The destabilized HeLo domain of HET-S then expels its N-terminal TM segment (residues 1–34, in red). (4) The exposed TM segment targets the activated HET-s/HET-S complex to the membrane where it is able to penetrate the membrane through the formation of a TM helix. The catalytic nature of the activation of HET-S by the HET-s amyloid suggests that activated HET-S may also be released from the co-aggregate before it enters the membrane. The HET-S membrane-disrupting oligomer may form as a direct result of its complex with the HET-s amyloid or only first as it inserts into the membrane. However, the membrane integrity is disrupted by hole-like structures, thus triggering cell death. The model for the HET-s fibril was created from the PFD fibril structure [17] and the HeLo domain structure [14] with an unwinding the last three helices of the HeLo domain (residues 177–222) to make space for the HeLo domains around the fibril. The HET-s HeLo domains are depicted as dimers between adjacent monomers in the fibril, but these are speculative and it should be emphasized that the structures of the HeLo domains of HET-s and HET-S, in the context of a fibril, are not known except that they lose tertiary structure (more molten globule-like), with a local loss of secondary structure around residues 190–220 [19], indicated, in the figure, by the spheres around the HeLo domains.

Here we show that HET-S and HET-s variants that give the [Het-S] phenotype can be activated in vitro by the HET-s prion to form a membrane-permeabilizing complex (Figures 1, 2, and 7). Our findings give detailed insights into prion-mediated heterokaryon incompatibility and support a new model for the underlying mechanism. Building upon our previous model for the HET-S prion-inhibition mechanism, our model for the mechanism of HET-S toxicity is depicted in Figure 7. HET-S interaction with HET-s prion aggregates leads to a template driven folding of the HET-S PFD into the same β-solenoid structure as the HET-s PFD (Figure 4). Due to the structural overlap of the HeLo and PFD domains, this folding of the PFD leads to a destabilization of the HeLo fold (Figures 4 and S2). This destabilization activates the prion-inhibitory activity as well as the toxic activity of HET-S. The destabilized HeLo domain of HET-S undergoes a restructuring that exposes the previously buried amino-acid residues of its TM helix, targeting the activated HET-S to the cell membrane where it assembles into a membrane-disrupting complex. We propose that HET-S activity is dependent on two of its functions: (i) membrane binding as demonstrated by the liposome pulldown assay (Figure 1), and (ii) oligomerization into the toxic entity as demonstrated by the MALS data with HET-S in membrane-mimicking detergents (Figure 6) and as suggested by the size of the defects in the liposome membranes (Figure 1). Interfering with either of these functions converts HET-S to a HET-s-like protein. The D23A mutation in HET-S eliminates the predicted TM helix and converts it to a more HET-s-like protein (Figure 5). The E86K mutation disrupts a dimerization interface that we previously identified in the HeLo domain [14] and therefore affects the oligomerization properties of HET-S and correspondingly its liposome leakage potential (Figure 3B). The H33P mutation does not eliminate the predicted TM helix (Figure S3); however, the proline may restrict the conformation of the helix so that it cannot oligomerize into the toxic entity. Other HET-s-like variants of HET-S although not studied here can also be classified into the type of function that they have likely lost. For example, HET-S[F25S] loses the predicted TM helix and becomes like HET-s [21]. A construct that fuses the HeLo domain to the PFD via a six-residue linker (relieving the domain overlap) has been shown to be HET-s-like [14], and this can be explained by the uncoupling of the HeLo domain structure from the aggregation of the PFD (Figures 4 and S2). Finally, all mutations in the PFD that disrupt the β-solenoid structure are null mutants. That this is true for both HET-S and HET-s further validates the model that the activation of HET-S toxicity involves a template-directed structuring of its PFD by the HET-s PFD.

### Discrepancies between In Vivo and In Vitro HET-S Activity

The proposed model in which HET-S is activated by a HET-s prion in order to carry out its toxic membrane-permeabilizing activity (Figure 1) may seem at odds with our results showing that HET-S can bind to and cause leakage from liposomes in a prion-independent manner (Figure 3) and that expression of HET-S alone leads to a growth arrest in *E. coli* (Figure 2). Taken at face value, these results contradict our model; however, it is easy to see how an in vitro or non-native in vivo system could lead to such discrepancies as discussed in the detail in Text S1. The most important factor described therein is that the mechanism of interest is dependent on changes in the thermodynamic stability of the HeLo domain, an event that in vivo is carefully orchestrated by the proteostasis including chaperones. While these apparent discrepancies can only be rationalized by arguments, it must be noted that there is a perfect correlation of the phenotype to the in vitro activity of the proteins (whether prion-induced or thermodynamically controlled) indicating the discussed activity is biologically relevant.

### An Amyloid Fold as a Conformational Activation Switch

Initially described in the context of human disease, amyloids were later found to be able to ensure a variety of functional roles for instance as cell surface structures in microbes or as storage and release devices of peptide hormones [2],[34],[35]. In the present model, the β-solenoid fold of HET-s is used as a specific conformational switch (Figure 7) for the activation of the pore-forming HeLo domain. This mode of activation is specific and simple as it does not involve a covalent modification such as a proteolytic cleavage or other type of post-translational modification. The folding energy associated with the acquisition of the amyloid structure drives the conformational conversion of the HeLo domain (Figures 4 and S2). This mode of regulation appears highly efficient, as very little HET-s seeds are required for conversion. Our study reveals that an amyloid fold can have a regulatory role in controlling the activity of another protein or domain, thus expanding the repertoire of functional tasks that can be assigned to amyloids. It appears that the inherent templating ability of amyloids is specifically exploited in this system not only to ensure maintenance of the [Het-s] state but also to allow for activation of HET-S by HET-s.

Since, the HET-s/HET-S system involves a prion amyloid in the context of a cell-death reaction, it was at first tempting to imagine that the mechanisms involved might be related in some way to the amyloid toxicity seen in human diseases. The present study now clarifies that this is not the case. It is not that amyloid toxicity is exploited to control a purposeful cell death reaction, but instead that the amyloid prion fold has a distinct functional role as an activation trigger of a toxic domain (Figure 7). The β-solenoid fold of HET-s is the trigger that converts the HET-S protoxin into a toxin (Figures 1C and 1D, 4, and 7). Our observations illustrate how both components of this cell death system can be harmless on their own but lethal when combined (Figure 1C and 1D). HET-s can constitutively harbor the β-solenoid fold because conversion of its own HeLo domain does not lead to toxicity while HET-S is stable in vivo as a protoxin while awaiting activation.

### Parallels between HET-S/s Prion, Mammalian Prion, and Amyloid Toxicities

The HET-s/S proteins, the yeast prion proteins Ure2p and Sup35 and the mammalian prion proteins (PrPs) are not homologous proteins and have no sequence similarities. What they have in common is the ability to transmit a phenotype via propagation of structural rearrangements in an infectious manner. However, they also share the structural motif of a globular folded domain attached to a highly flexible region that is able to convert to an amyloid-like entity [36]. In contrast to HET-s with a folded N-terminal HeLo domain, the folded domain of PrP (residues 124–227) is C-terminal to its flexible domain (residues 23–123). Further similarities are that for both HET-s and PrP, the prion forming domains (residues 218–289 in HET-s and ∼23–140 in PrP) overlap with the folded domain, thus requiring a conformational change of the latter upon prion formation. In addition, the globular domains of HET-S and PrP have a predicted TM helix (residues 1–34 in HET-S and 113–128 in human PrP) that is integrated into the soluble fold (Figures S3 and S4 for HET-S). These similarities that occur in unrelated prion systems are intriguing and let us speculate that like in the HET-s/S system (Figure 7), conversion of PrP^C^ to the prion form PrP^Sc^, may induce a structural transition in its globular domain that exposes a TM segment, thus forming an integral membrane protein that exerts its toxicity through membrane interaction. This hypothesis is supported by some experimental data showing that PrP^C^ (including in particular familial variants thereof) can be an integral membrane protein under certain circumstances associated with toxicity [37].

While we have observed that the HET-S HeLo domain itself is toxic when overexpressed in *E. coli* (Figure 2), the PFD is also required for the HeLo domain to function in vivo. Therefore we cannot exclude that the amyloid structure of the PFD (Figure 4) is not intimately involved in the membrane disruption activity (i.e., more than as an oligomerization scaffold). There are many examples of amyloid proteins that can disrupt membrane integrity though channel or pore formation [38], most notably Aβ, which has been shown to form non-specific ion channels [39]. The propensity of amyloids to associate with lipid bilayers has led to the channel hypothesis of amyloid toxicity (reviewed in [40]). However, the similarity between HET-s and other pore-forming amyloids does not go beyond the fact that they are both amyloids. Our data currently support a pore-forming activity in the HeLo domain with a mode of activation that more resembles larger non-amyloid cytotoxins than pure amyloid peptide-based toxicity (Figure 7).

### HET-S Is a Pore-Forming Toxin

The mechanism of HET-S toxicity is general enough to be organism-independent (observed in bacteria [Figure 2], yeast, and filamentous fungi) and involves a membrane association. As we demonstrate, HET-S forms holes in membranes and causes the leakage of liposomal contents (Figure 1). HET-S should therefore be considered a pore-forming toxin. Pore-forming toxins are a large class of virulence factors employed by a wide range of bacterial pathogens (such as diphtheria toxin and anthrax toxin) but are also found in eukaryotes where they can be involved in immune defense [41]. As implied by their name, this class of toxins forms pores in the membranes of target cells that result either in membrane leakage or in the delivery of toxic components through the pores. They are a diverse class of proteins that are further classified as α- or β-pore-forming toxins, depending on the type of secondary structure they acquire when inserted into the membrane. However, despite the wide diversity in sequences and structures of pore-forming toxins, they typically act by a common mechanism. One important characteristic of pore-forming toxins is their transformation from a soluble, monomeric protein to an integral membrane protein oligomer [42],[43]. For this transformation the toxin must undergo a conformational change in which hydrophobic segments that are buried in the soluble fold become exposed to the hydrophobic environment of the membrane lipids. In this manner, many toxins can be secreted in a soluble form that diffuses to the membrane of the target cell. Oligomerization of the toxin initiated by another protein/antigen/small molecule to a prepore is necessary for stable binding to the target cell and for membrane integration [44],[45]. HET-S therefore bears many of the typical characteristics of pore-forming toxins. In addition to the toxicity induced by its membrane association and the generation of pore-like entities in liposomes (Figure 1), it also has a protein partner that triggers the conformational change from a soluble to an integral membrane protein (i.e., the HET-s prion) (Figures 1, 2, and 4). While the structure of the membrane active complex has yet to be determined for HET-S, the fact that it has a predicted TM helix and the observation that the corresponding peptide fragment in detergents is helical as determined by CD (unpublished data) makes it likely to be an α-pore forming toxin.

Whether as a mechanism of defense or attack, production of pore-forming toxins is an ancient and conserved mode of warfare between cells. The majority of the currently characterized pore-forming toxins are of bacterial or animal origin [46]. Yet, the existence of pore-forming toxins has also been reported in the fungal kingdom where they function in pathogenicity, host-defense, and inter-organismal competition [47]–[50]. It is striking that the cell death process occurring in heterokaryon incompatibility also appears to rely on that basic and ancient mode of toxicity based on membrane disruption.

### Implications for Fungal Incompatibility

This study represents the first insight into the mechanistic aspects of cell death by incompatibility in any fungal system. It appears that in this system induction of cell death occurs by a direct and universal toxicity mechanism and relies on the formation of a protein structure related to pore-forming toxins. Pore-forming toxins are typically used in prokaryotes and eukaryotes both as attack and defense molecules in various pathogenic or competitive biotic interactions. One may find it surprising that an organism should use this mode of toxicity against itself by targeting its own membrane as occurs in HET-s/HET-S incompatibility. It has been proposed that genes controlling heterokaryon incompatibility in *Podospora* may derive by exaptation from genes involved in host-defense against heterospecific non-self [51]. The HET-S/HET-s system could exquisitely illustrate this concept. Indeed, the mechanism of HET-S toxicity induction postulated here might also occur in a different context. Recently, a potential additional functional partner of HET-S was identified. A search for proteins displaying homology to the HET-s PFD has identified a protein termed NWD2 encoded by the gene immediately adjacent to *het-S* in the genome of *Podospora* [52]. NWD2 belongs to the NWD-gene family comprising other *het*-genes. NWD proteins are STAND proteins resembling Nod-like receptors and are believed to represent the fungal counterparts of pathogen recognition receptors described in plants and metazoans. STAND proteins are signal-transducing NTPases that undergo ligand-induced oligomerization and typically display three domains: a central nucleotide-binding oligomerization domain (NOD) flanked by a C-terminal ligand-binding domain and an N-terminal effector domain. NWD2 lacks a defined effector domain and in place displays at the N-terminal end a short region of homology with the elementary repeat motif of the HET-s PFD. A model postulating the existence of a functional interaction between NWD2 and HET-S was proposed. In that model, NWD2 recognizes a non-self ligand via its C-terminal WD-40 repeat domain and oligomerizes in response to this binding. This oligomerization step would put the N-terminal extensions of NWD2 proteins into close proximity and allow their cooperative folding into the β-solenoid fold. Once formed this fold would template the HET-S PFD thus activating the HeLo toxicity domain much in the same way as the HET-s prion does. In this mode of activation, it might be that the membrane-disrupting activity of HET-S is used as part of a host-defense or attack mechanism and directed to the membrane of a biotic interactant of *Podospora*. This second proposed mode of activation appears more widespread and evolutionary ancient than the HET-s-induced activation, because the *het-S nwd2* gene pair is conserved in a number of distant species while there is no evidence for the existence of *het-s/het-S* incompatibility system outside of *Podospora*. Since a simple loss of function mutation in the HeLo domain (for instance mutation H33P) will turn HET-S into a non-toxic prion protein that now acquires the ability to trigger activation of HET-S, it is easy to see how the HET-s/HET-S might have evolved by exaptation starting from *het-S*.

### Domain Architecture and TM Helix Conservation in HeLo Domain Proteins Allude to a Large Family of Pore-Forming Toxins in Filamentous Fungi

**Figure 8:**
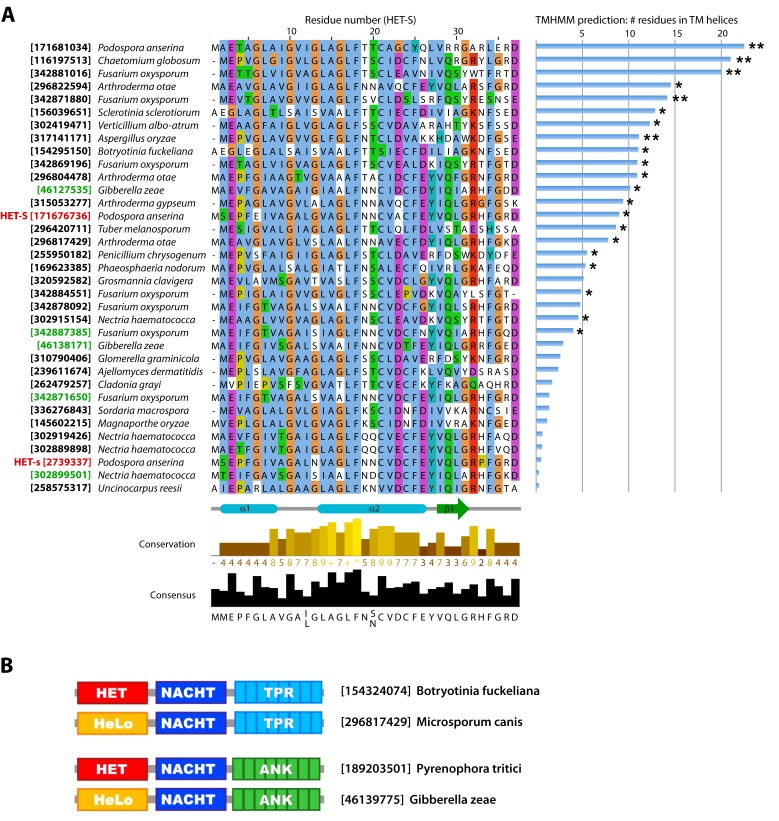
HeLo domain alignment of the TM segment and HeLo/HET domain architecture comparison. (A) The sequences in this alignment are the non-redundant output of a PSI-BLAST search with residues 4–33 of HET-S (carried to convergence at an E-value threshold of 0.005). Therefore, these 35 sequences are a subset of HeLo domains that are more similar to HET-S in their TM region. The output score of the TMHMM algorithm (sum of per-residue probability) is plotted to the right of the sequences with a double asterisk to indicate those that are predicted to have a TM helix. When only residues 1–38 are input into the algorithm many more (21 of 35) sequences are predicted to have a TM helix (indicated by a single asterisk). The GI accession numbers in red are for HET-S and HET-s and those in green are for the sequences which have the HET-S domain architecture (HeLo-PFD). (B) Two examples of HeLo domain-containing STAND proteins that have similar architectures to HET domain-containing STAND proteins. The known role of the HET domain as an inducer of cell death suggests that the HeLo domain may have a similar role.

There are to date more than 100 HeLo domain sequences retrieved with an iterative PSI-BLAST search, all of which belong to the genomes of filamentous fungi (Pfam accession: PF14479). Among those are putative *het-S-*homologs with an associated PFD, all of which are adjacent in their respective genomes to a gene encoding a STAND protein with a consensus PFD-like motif at its N-terminus [52]. A number of HeLo domains occur as N-terminal domains of STAND proteins. This domain organization can be seen as the “all-in-one” counterpart of the *het-S nwd2* gene pair architecture [52] and is analogous to the three domain architecture found in other PCD inducing *het* genes from *Podospora*, in which the HeLo domain is replaced by another type of cell-death–inducing domain termed the HET domain (Figure 8B). Since the HET domain has been shown to mediate PCD [53] and HeLo and HET domains both occur as the N-terminal domain of numerous STAND proteins, we suggest it is likely that the HeLo domain has a general role as a cell death-inducing domain. Our data indicate that the HET-S HeLo domain mediates PCD through its action as a pore-forming toxin upon activation through oligomerization of its C-terminal PFD. This finding is also consistent with the fact that in plant and animal kingdoms, STAND proteins have been shown to oligomerize, leading to activation of their N-terminal effector domain [54],[55], such as the death domain, the death effector domain, or the caspase-activation and recruitment domain [56].

In addition to the STAND proteins, the HeLo domain appears in many proteins with uncharacterized domains (domains that are automatically generated by ADDA [57]). Interestingly, the HeLo domain is found almost exclusively at the N-termini of the proteins and the few exceptions could be artifacts of the automatic ORF prediction and annotation of the sequence databases. That the HeLo domain appears as the first domain in multi-domain proteins further suggests that it has a conserved function involving the insertion of its own N-terminal segment into a lipid membrane. With it being an N-terminal domain there are no topological restrictions on its insertion in to the membrane, whereas a centrally located HeLo domain would need either an extra TM segment or the ability to translocate the domains N-terminal to it, across the membrane. Furthermore, the TM segment is well conserved in HeLo domain proteins as highlighted in Figure 8. The sequences in this alignment are the non-redundant output of a PSI-BLAST search with residues 4–33 of HET-S (carried to convergence at an E-value threshold of 0.005). Therefore, these 35 sequences are a subset of HeLo domains that are more similar to HET-S in their TM region. The output of the TMHMM algorithm, represented graphically in Figure 8, indicates that 21 of the 35 sequences have a TM helix (when only residues 1–38 are analyzed, see Figure 8 legend) while another algorithm, TMPRED, predicts all of them to have TM helices except for HET-s, which is classified as “putative.” The prediction of a high conservation of the TM helix is not simply an artifact of our having performed the PSI-BLAST search with the TM region of HET-S. TMPRED identifies the same TM helix in nearly all of the more than 100 HeLo domains in the sequence databases. We therefore conclude that the conservation of the TM helix as well as the HeLo domain's context and its N-terminal position in the sequence of multidomain proteins indicates that HeLo domain proteins are a widespread (in filamentous fungi) class of pore-forming toxins.

## Methods

### Plasmids and Protein Constructs

Constructs with N-terminal histidine tags were expressed from genes that were sub-cloned into the pRSET vector, yielding a protein product with the N-terminal sequence MRGSHHHHHHGLVPRG/S directly preceding the coding region. Thus cleavage of the protein product with thrombin (recognition site underlined, cleavage indicated by a backslash) yielded the protein of interest with an N-terminal serine. The C-terminally histidine-tagged constructs were expressed from pET21 or pET24 vectors with the tag directly following the last residue of the protein (no cleavage site).

### Protein Expression and Purification

Cultures of *E. coli* BL21*(DE3)pLysS were prepared from a single fresh transformant in the following manner: a single colony was streaked onto a new LB plate, grown overnight at 37°C, and then the entire plate was used to inoculate M9 minimal medium at a starting OD_600_ of 0.1–0.2. When the cell density reached OD_600_ = 0.8, the culture was transferred to 18°C and protein expression was induced with 0.5 mM IPTG for 12 h. All the mutants were expressed similarly, but for 6 h at 27°C. The cells were collected by centrifugation and re-suspended in the lysis buffer (50 mM Tris pH 8.0, 200 mM NaCl, 20 mM imidazole, 2 mM DTT, 10% v/v glycerol, 1 mM EDTA) with 1 complete, EDTA-free Protease-Inhibitor Cocktail tablet (Roche) per 50 ml of Lysis buffer and 0.5 mg/ml lysozyme (Sigma) at 4°C. Lysis was achieved by three passes over a microfluidizer at processing pressure of 18,000 psi (M-110S, Microfluidics) and the insoluble material was removed by centrifugation at 100,000 *g* for 35 min. The supernatant was passed over a nickel FF-sepharose column (GE Healthcare) that was then washed with ten column volumes lysis buffer followed by elution buffer (50 mM Tris pH 8.0, 200 mM NaCl, 200 mM imidazole, 2 mM DTT, 10% v/v glycerol). The histidine-tag was removed by thrombin (bovine plasma, Sigma) treatment at room temperature overnight in the elution buffer. Thrombin was removed on a benzamidine FF column (GE healthcare). The protein was further purified on a Superdex 200 gel filtration column (GE Healthcare) pre-equilibrated with 10 mM NaPO_4_ (pH 7.4), 200 mM NaCl, 10% glycerol, 1 mM DTT. The protein was either used immediately or frozen in small aliquots in liquid N_2_ and stored at −80°C.

HET-s(218–289) and HET-s variants were expressed as described for other HET-s constructs [10] and therefore the purification procedure is only described briefly. The bacterial pellet was lysed in lysis buffer, passed over a microfluidizer and the insoluble material was collected as described above. The pellet was then solubilized in 50 mM Tris (pH 8.0), 150 mM NaCl, 6 M guanidinium hydrochloride overnight at 60°C. After centrifugation for at least 2 h at 150,000 *g*, the protein was purified from the supernatant using a nickel FF-sepharose column and then desalted into 200 mM acetic acid. Monomeric protein was immediately lyophilized and stored at −80°C. Full-length HET-S with a non-cleavable C-terminal His-tag was also expressed and purified as follows: one plate of transformed cells was pooled into a starter culture of LB medium and grown at 37°C to an OD_600_ of 0.6, pelleted at 5,000 *g*, and resuspended in 1 l minimal medium with a starting OD_600_ of ∼0.1. At an OD_600_ of 0.6, the culture was induced with 0.5 mM IPTG for 3–4 h at 37°C. This protein construct was purified as described above from the soluble fraction, but it was also partially present in the membrane fraction of *E. coli*. For the membrane fractionation, cells were lysed by three passes over a microfluidizer at processing pressure of 18,000 psi. The protein lysate was centrifuged at 8,000 *g* for 15 min to collect non-lysed cells and large debris. The supernatant was immediately subjected to a 2-h centrifugation at 100,000 *g*. The pellet containing the membranes was resuspended in PBS containing 1 M Urea. Washed membranes were harvested subsequently by a second round of centrifugation. This crude membrane fraction was treated overnight with 1% N-laurylsarcosine in PBS and then centrifuged again at 100,000 *g*.

### Protein Expression and Cell Density

Cultures of *E. coli* BL21*(DE3)pLysS were prepared from transformants of an entire plate. All colonies were resuspended in 10 ml of LB media, the OD was measured, and cells were diluted in 100 ml to a starting OD_600_ of 0.1–0.2. The cultures were incubated at 37°C with shaking, and when the cell density reached OD_600_ = 0.5, they were induced with 0.5 mM IPTG for 7 h. During the entire growth, the OD_600_ was measured every 30 min.

### Membrane Fractionation with a Sucrose Density Gradient

The separation of inner and outer membranes of *E. coli* was adopted from a protocol described by Osborn et al. [23]. Briefly, cells from a 4 h induction of HET-S (C-terminal HisTag) in LB medium at 37°C were resuspended at 0.2 g wet-cell-weight per ml of lysis buffer (including protease inhibitor and lysozyme) and sonicated in a volume of 2 ml with a Bandelin Sonopuls UW2070 microtip at 70% power for 1 min on ice. The lysates were centrifuged for 5 min at 5,000 *g* at 4°C to remove unbroken cells. The supernatant (1.2 ml) was mixed with 50% (w/w) sucrose (0.8 ml) so that the resulting mixture was 25% (w/w) sucrose, and 1 ml of this was loaded onto the top of the sucrose gradient. The sucrose gradient was prepared in a 12-ml centrifugation tube (Beckman, REF 331372) by layering 2.4 ml of 50%, 1.8 ml each of 45%, 40%, 35%, and 2.3 ml 30% over a 0.5-ml 55% sucrose cushion. All sucrose solutions contained 10 mM Tisi, X mM EDTA (pH 8). The gradients were centrifuged in a SW41 rotor at 30,000 rpm for 16 h at 4°C. Fractions of 1 ml each were collected though a needle by inserting it from the top so that the tip was 5 mm from the bottom of the tube. The bottom 200 µl (Figure 3, fraction 1) were removed afterwards with a pipette so as to retrieve any material that sedimented through the 55% cushion onto the bottom of the tube. DTT (2 mM) was immediately added to the fractions and they were separated by SDS-PAGE and visualized by both Coomassie staining and Western blot with an anti-HisTag antibody (Abcam 18184) using a standard ECL kit (GE Healthcare). The NADH oxidase activity of each fraction was measured in triplicate by mixing 50 µl with 350 µl water and then 400 µl NADH buffer (100 mM Tris pH 7.5, 0.2 mM DTT, 0.12 mM NADH). The rate of decrease of absorbance at 340 nm during the first 5 min was taken as the relative activity of NADH oxidase.

### Formation of Coaggregates for Solid-State NMR Studies

Lyophilized monomeric HET-s(218–289) was solubilized by an appropriate amount of 1.5 M Tris (pH 8) to reach (pH 7) followed by immediate mixing with HET-S (10 mM NaPO4, 200 mM NaCl, 1 mM DTT) at a 1∶1 molar ratio. The co-aggregating mixtures were incubated with slow rotation in 50 ml tubes for at least 7 d at room temperature. The fibrils for solid-state NMR were centrifuged directly into a 3.2-mm Bruker MAS rotor at 200,000 *g* using a custom-made tool [58].

### Preparation of Liposomes with and without Calcein

*E. coli* –derived polar lipids were purchased from Avanti Polar Lipids. Chloroform-containing lipid solutions were dried in round bottom flasks (8 cm in diameter) under a gentle nitrogen stream in a fume hood. Residual chloroform was removed under vacuum. For the calcein release assay, the dry lipid film resulting from evaporation was resuspended in aqueous 60 mM calcein (Sigma), which was prepared in 10 mM Tris, 150 mM NaCl. The pH of the calcein solution was adjusted with concentrated 1 M Tris to (pH 8) and the solution was filtered through a 0.2-µm membrane before it was added to the dried lipids. The resuspended solution at a final lipid concentration of 20 mg/ml was incubated for 1 h at room temperature with occasional vortexing to allow lipid hydration and vesicle formation. After 1 h, the lipid suspensions were vigorously vortexed to allow complete detachment of hydrated lipids. The resulting suspension was subjected to five freeze-thaw cycles of 10 min each: freezing in liquid nitrogen followed by 10 min thawing at 37°C. Calcein-loaded vesicles were frozen at a concentration of 20 mg/ml and in 100 µl aliquots at −20°C. For the preparation of liposomes for cryo-EM, PK digestion, and liposome binding assays, the lipid film was resuspended in buffer L (20 mM Tris, 150 \mM NaCl, pH 7.5) without calcein and then processed as in the calcein-containing lipids.

### Calcein Leakage Assay

Liposomes for the calcein leakage assay were prepared starting with 1 ml of calcein-loaded vesicles that were diluted to a lipid concentration of 2 mg/ml and extruded with 20 passes through a 400-nm porous membrane (Whatman), followed by 20 passes through a 100-nm membrane. The calcein that remained outside of the liposomes was separated from extruded liposomes by two sequential passes over Sephadex G-25 PD10 columns (GE Healthcare) pre-equilibrated with buffer L. The calcein-loaded liposomes were then diluted 100-fold for the calcein leakage assay and used within 2 d. Liposomes were mixed with wild-type HET-S and HET-s or their variants as indicated in the text at final concentrations in a range between 8 µM to 80 nM. The assay was carried out using a plate reader instrument (Pherastar, BMG) and 96-well plates (Greiner Bio, 655900) allowing for simultaneous data acquisition of 32 reactions in triplicate (including the control samples). The release of calcein from liposomes was monitored over time as an increase of calcein fluorescence intensity (λ_ex_ = 485 nm; λ_em_ = 520 nm). Leakage was calculated from average values of the triplicate measurements following fthe equation 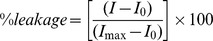, where 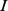 and 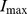 are the emission intensities in buffer controls and in liposome solutions with the addition of 1% SDS, respectively.

The standard deviation of the leakage was calculated in three steps. First, the standard deviation of each measured intensity was calculated from 
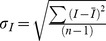
, where 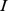 is the value, 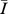 is the average value, and 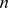 the number of values, here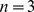. Second, the standard deviations of the blank-subtracted values 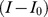 and 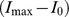 were calculated from standard deviations of the sample and blank intensitiesstandard deviations: 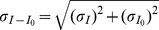. Third, the standard deviation of the leakage was calculated: 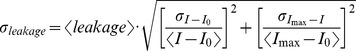 where the angled brackets indicate average values.

### Freeze-Fracture EM of Liposomes

For the cryo-EM studies, 100 nm extruded liposomes (10 mg/ml) were incubated with and without 20 µM protein at 37°C or 4°C for 90 min. Droplets of the liposome solution were placed on an Au-grid between two copper blades used as sample holders and then frozen in liquid propane cooled to −180°. Freeze fracturing of the samples was performed in a Balzers 400T apparatus cooling the specimen stage at −160°C. Etching occurred at −110°C for 8 min, then Pt/C shadowing was applied in a 45° angle (with respect to the specimen stage) and pure carbon at 90° onto the sample. After thorough cleaning of the replicas in water, EtOH and Acetone, Au-grids were analyzed in a FEI Morgagni TEM electron microscope at 100 kV with nominal magnification of 8,000×, 16,000×, 32,000×. The freeze fractures were also analyzed by a Zeiss 1530 Gemini at 5 kV with nominal magnifications up to 200,000× at −120°C.

### Liposome Pull-Down Experiments

Proteins were mixed with 20 µl of 20 mg/ml *E. coli* polar liposomes or buffer L. The volume of all samples was adjusted with buffer to 50 µl and samples were incubated at 30°C for 2 h or overnight or at 4°C overnight. After incubation liposomes were pelleted by a 20-min centrifugation at 180,000 *g*. Control samples containing no liposomes were treated alike as a control for liposome-independent protein precipitation. The supernatant was immediately collected and mixed with SDS-PAGE-loading buffer. The pellet was resuspended in 50 µl PBS containing 0.1% DDM and 1× SDS-loading buffer. Samples were directly subjected to SDS-PAGE or immediately frozen at −20°C.

### Proteinase K Digestion

50 µg of HET-S protein were digested on ice for 30 min with 20, 2, 0.2, and 0.02 µg of PK in a volume of 250 µl after incubation at 37°C for 1 h in the presence and absence of 16 mg/ml liposomes. Reactions were stopped by mixing equal volumes of the reaction and 30 mM phenylmethylsulfonylfluorid (PMSF). Liposomes were separated from the supernatant by centrifugation at 180,000 *g* for 10 min. 40 µl of the supernatant was mixed with a 6× SDS loading buffer and boiled (final 2× SDS loading buffer) for 5 min. The pellet was first resuspended in 40 µl of buffer and then brought to 2× SDS loading buffer in the same manner. 20 µl of each reaction were immediately analyzed by SDS-PAGE followed by Coomassie Blue staining. For N-terminal sequencing, an unstained SDS-PAGE copy was transferred onto a PVDF membrane and analyzed by the Functional Genomics Center Zurich.

### Circular Dichroism Spectroscopy

Concentrated protein constructs were diluted to 20 µM in 0.4% FC-12, PBS, 10% glycerol for analysis on a Jasco J-815 CD spectrometer. Far UV CD spectra (190–240 nm) were measured at 27°C with a 20-nm/min scan rate in a 0.1-cm path-length cuvette. The loss of structure in HET-S was observed for 16 h by collecting spectra every hour.

### Size-Exclusion Chromatography

Recombinant HET-S and HET-S 227 were mixed with 0.4% of FC-12, PBS, 10% glycerol to a final concentration of 20 µM protein. The protein solutions were separated by size with a G4000PW_XL_ gel filtration column (Tosoh Bioscience) in the same buffer on an Agilent 1200 series HPLC system injected by the autosampler (20 µl) at a flow rate of 0.5 ml/min. The column eluate was simultaneously monitored by the 1,200 series diode array detector, a TREOS light-scattering detector (Wyatt Technology), and the 1,200 series refractive index detector. The signals from the detectors were analyzed by protein conjugate analysis in the Astra V program (Wyatt Technology) using an average protein dn/dc value of 0.187 ml/g, the literature value for FC-12 (0.1398 ml/g) (www.wyatt.eu) and the protein extinction coefficient as calculated by the ProtParam tool (http://web.expasy.org/protparam/).

### Solid-State NMR

All solid-state NMR spectra were recorded on a Bruker Avance II+ 850 spectrometer operating at a static magnetic field of 20.0 T using a Bruker 3.2 mm triple-resonance low-E (LLC) magic-angle spinning (MAS) probe. The experiments were carried out at MAS frequencies of 17.5 or 18 kHz at sample temperatures of 3–10°C. All experimental parameters are given in Table S2.

## Supporting Information

Figure S1 **HET-S in the presence of HET-s(218–289) amyloid seeds makes holes in liposomes observed by freeze-fracture electron microscopy while HET-S alone does not.** (A) Cryo-SEM (scanning electron microscopy) images of freeze fractures of 100 nm diameter extruded liposomes incubated in the presence of a mixture of HET-S and HET-s(218–289) fibril seeds. These images show membrane damage. (B) For negative control cryo-SEM images of liposomes incubated at 4°C in presence of HET-S only.
(PDF)

Figure S2 **Aliphatic region of the DARR solid-state NMR spectrum (100 ms mixing) of HET-S aggregates (blue) and HET-s(218–289) fibrils (black contours).**
(PDF)

Figure S3 **TM helix predictions (output of TMHMM with residues 1–33 as input) are shown for several interconverting variants of the TM region.** The genotype of each construct is in italics and its phenotype is in square brackets. The histograms are the per-residue probability output from TMHMM with the [Het-S] phenotypical proteins in grey and the location of the TM segment (if predicted) indicated by a brown bar. There is a good correlation between the prediction of a TM helix and the HET-S phenotype and the only exception is HET-S[H33P], which is discussed in the text. The residues that are replaced in the variants are labeled by a star and the single letter code of the substituted amino acid. The graphs on the left are HET-S and its variants with the wild type HET-S sequence below and those to the right are of HET-s and its variants. The differences between the two wild type sequences are highlighted in red. The [Het-S^s^] phenotype is an unstable [Het-S] that spontaneously or after contact with [Het-s] converts to [Het-s] [24].
(PDF)

Figure S4 **Sequence alignment of HET-s and HET-S.** The 13 out of 289 not identical residues are spread over the whole protein and are highlighted in red. Both proteins consist of a globular N-terminal HeLo domain (residues 1–227) and a C-terminal PFD (residues 218–289, underlined in black). The secondary structural elements are indicated for the HeLo domain as present in its soluble state and for the PFD in its aggregated state. HeLo domain and PFD overlap by 10 residues (highlighted by a blue box).
(PDF)

Table S1 **HET-S/s variant phenotype correlation to calcein leakage and TM prediction.**
(PDF)

Table S2 **Experimental parameters of the solid-state NMR spectra.** Their pulse sequences are given in [26]. The DREAM transfer is described in [59].
(PDF)

Text S1 **Detailed discussion on the discrepancies between in vivo and in vitro HET-S activity.**
(PDF)

## Abbreviations

CD: circular dichroism
FC-12
NMR: nuclear magnetic resonance
PFD: prion-forming domain
PK: proteinase K
PrP: prion protein
TEM: transmission electron microscopy
TM: transmembrane
