# Supplementary figures and images for: The Mechanism of Toxicity in HET-S/HET-s Prion Incompatibility
Source: PLoS Biol. 2012 Dec 27;10(12):e1001451. doi: 10.1371/journal.pbio.1001451 (PMC3531502; doi:10.1371/journal.pbio.1001451)

**Figure S1**

**A**

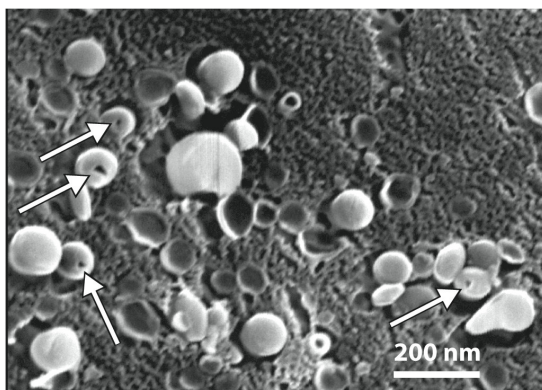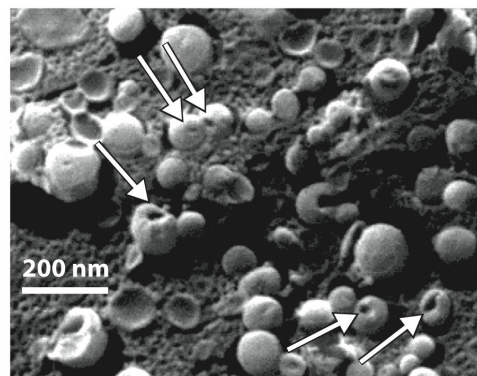

**B**

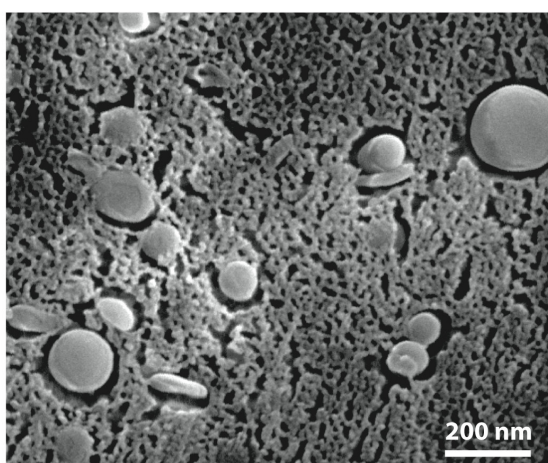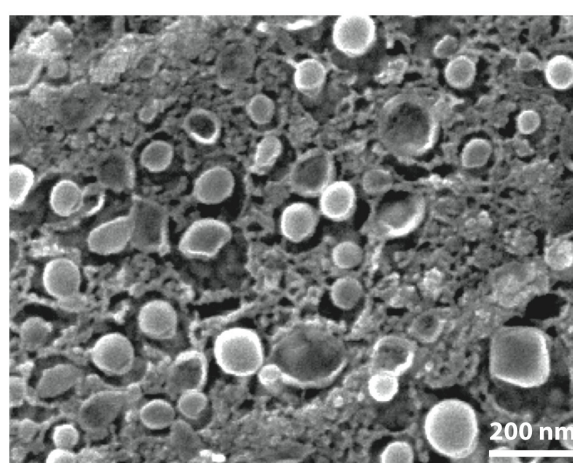

Supplement: Figure S1 — HET-S in the presence of HET-s(218–289) amyloid seeds makes holes in liposomes observed by freeze-fracture electron microscopy while HET-S alone does not. (A) Cryo-SEM (scanning electron microscopy) images of freeze fractures of 100 nm diameter extruded liposomes incubated in the presence of a mixture of HET-S and HET-s(218–289) fibril seeds. These images show membrane damage. (B) For negative control cryo-SEM images of liposomes incubated at 4°C in presence of HET-S only. (PDF) [file pbio.1001451.s001.pdf]

Figure S2

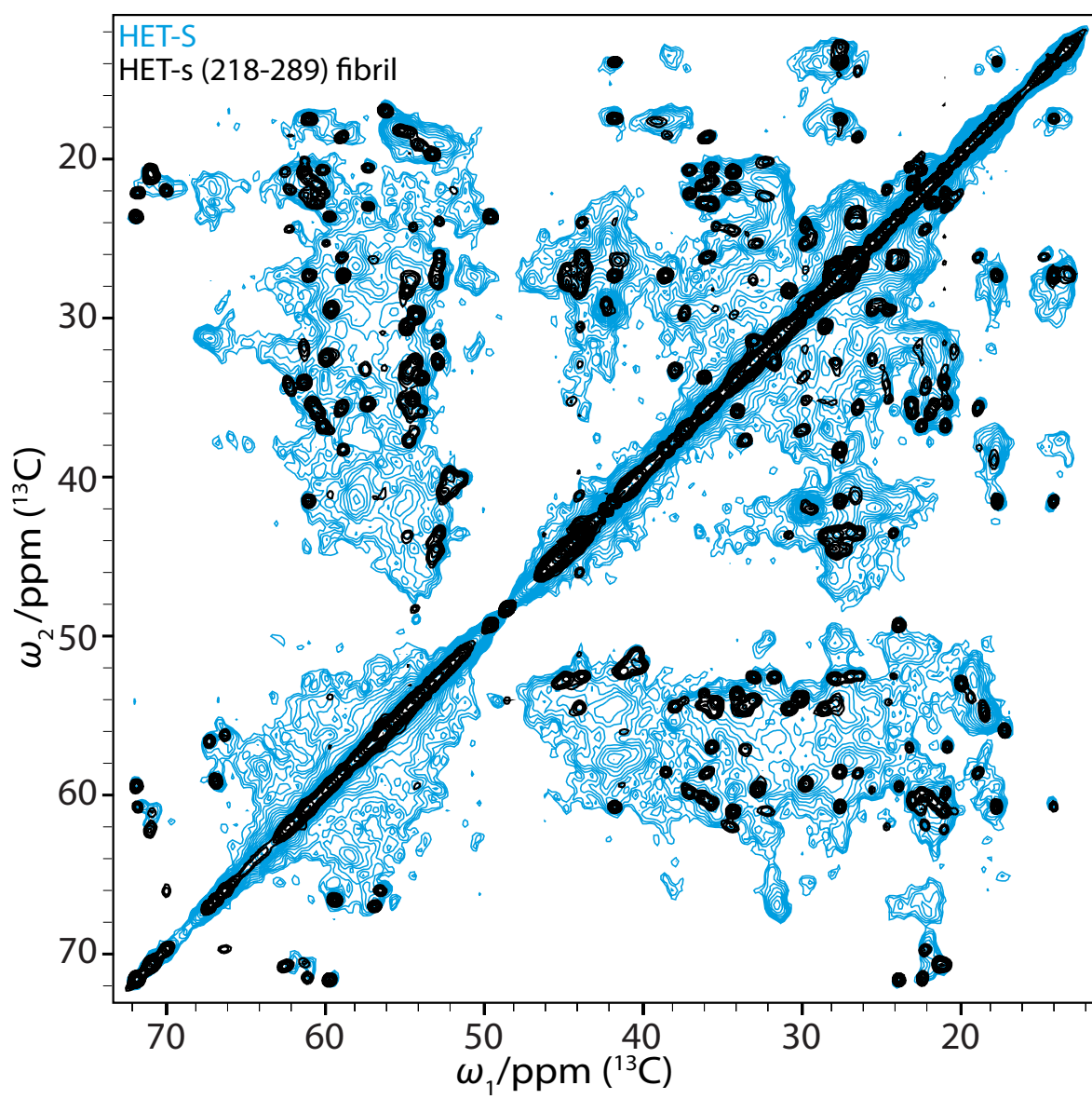

Supplement: Figure S2 — Aliphatic region of the DARR solid-state NMR spectrum (100 ms mixing) of HET-S aggregates (blue) and HET-s(218–289) fibrils (black contours). (PDF) [file pbio.1001451.s002.pdf]

Figure S3

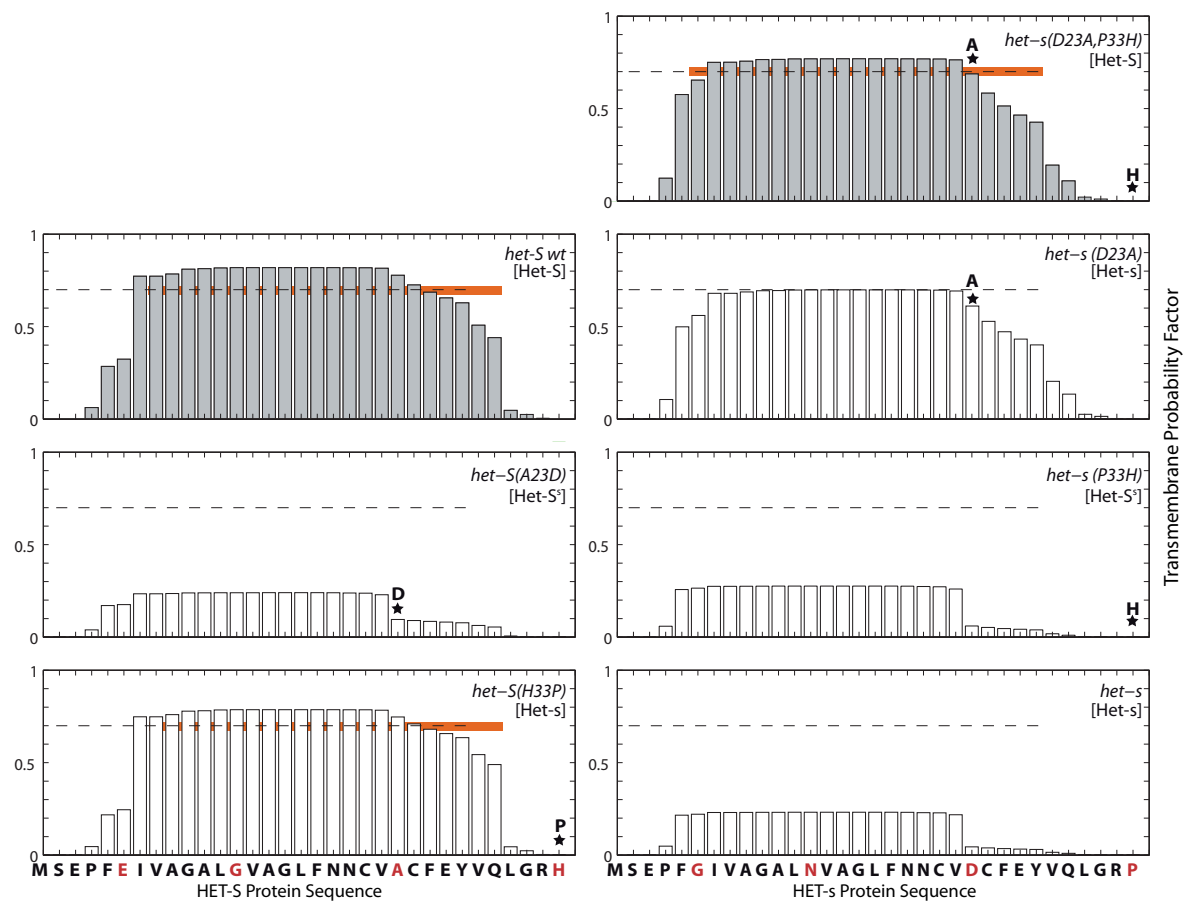

Supplement: Figure S3 — TM helix predictions (output of TMHMM with residues 1–33 as input) are shown for several interconverting variants of the TM region. The genotype of each construct is in italics and its phenotype is in square brackets. The histograms are the per-residue probability output from TMHMM with the [Het-S] phenotypical proteins in grey and the location of the TM segment (if predicted) indicated by a brown bar. There is a good correlation between the prediction of a TM helix and the HET-S phenotype and the only exception is HET-S[H33P], which is discussed in the text. The residues that are replaced in the variants are labeled by a star and the single letter code of the substituted amino acid. The graphs on the left are HET-S and its variants with the wild type HET-S sequence below and those to the right are of HET-s and its variants. The differences between the two wild type sequences are highlighted in red. The [Het-Ss] phenotype is an unstable [Het-S] that spontaneously or after contact with [Het-s] converts to [Het-s] [24]. (PDF) [file pbio.1001451.s003.pdf]

Figure S4

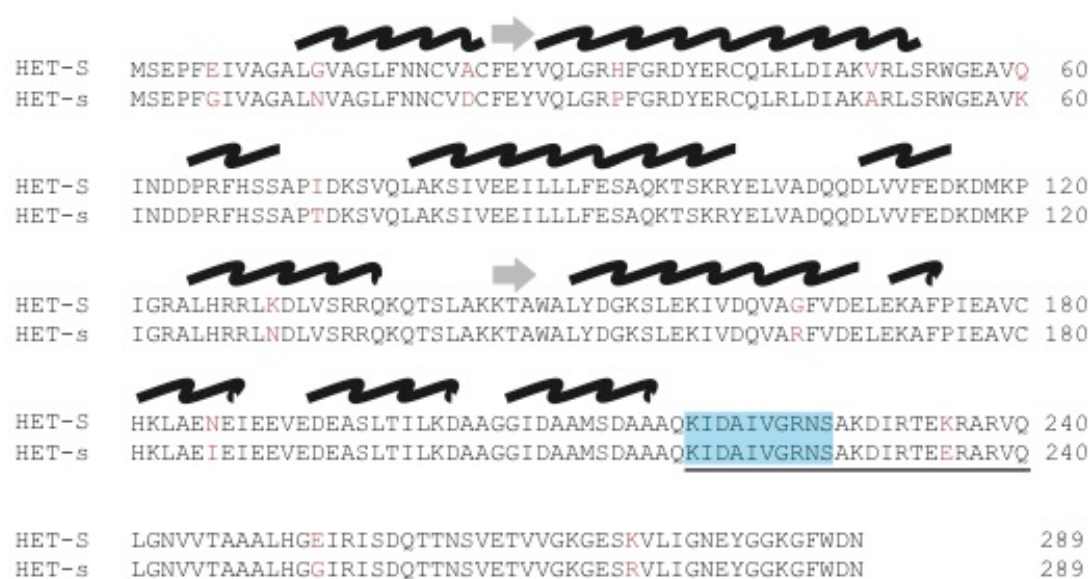

Supplement: Figure S4 — Sequence alignment of HET-s and HET-S. The 13 out of 289 not identical residues are spread over the whole protein and are highlighted in red. Both proteins consist of a globular N-terminal HeLo domain (residues 1–227) and a C-terminal PFD (residues 218–289, underlined in black). The secondary structural elements are indicated for the HeLo domain as present in its soluble state and for the PFD in its aggregated state. HeLo domain and PFD overlap by 10 residues (highlighted by a blue box). (PDF) [file pbio.1001451.s004.pdf]
