# Supplementary material for: The Mechanism of Toxicity in HET-S/HET-s Prion Incompatibility
Source: PLoS Biol. 2012 Dec 27;10(12):e1001451. doi: 10.1371/journal.pbio.1001451 (PMC3531502; doi:10.1371/journal.pbio.1001451)
Supplement: Table S1 — HET-S/s variant phenotype correlation to calcein leakage and TM prediction. (PDF) [file pbio.1001451.s005.pdf]

Table S1 HET-S/s variant phenotype correlation to calcein leakage and TM prediction.

Genotype	*het-S*	*het-S (A23D)*	*het-S (H33P)*	*het-s*	*het-s (D23A,H33P)*
Phenotype	[Het-S]	[Het-S^{s}][1]	[Het-s]	[Het-s]	[Het-S]
TM prediction[2]	+	-	+	-	+
Calcein leakage	+	-	-	-	+

[1] This phenotype is an unstable [Het-S] that spontaneously or after contact with [Het-s] converts to [Het-s][1].
[2] Results of the TMHMM algorithm predictions for residues 1-38 of the proteins.

References

1. Deleu C, Clave C, Begueret J (1993) A single amino acid difference is sufficient to elicit vegetative incompatibility in the fungus *Podospora anserina*. Genetics 135: 45-52.
