# Supplementary material for: The Mechanism of Toxicity in HET-S/HET-s Prion Incompatibility
Source: PLoS Biol. 2012 Dec 27;10(12):e1001451. doi: 10.1371/journal.pbio.1001451 (PMC3531502; doi:10.1371/journal.pbio.1001451)
Supplement: Table S2 — Experimental parameters of the solid-state NMR spectra. Their pulse sequences are given in [26]. The DREAM transfer is described in [59]. (PDF) [file pbio.1001451.s006.pdf]

Table S2 Experimental parameters of the solid-state NMR spectra.[1]

Sample	HET-s(218-289) fibril	HET-s(218-289) monomer + [13C,15N] HET-S monomer	HET-s(218-289) seeds + [13C,15N] HET-S monomer	HET-s monomer + [13C,15N] HET-S monomer	15N HET-s(218-289) monomer + 13C HET-S monomer
Spectrum	NCA	NCA	NCA	NCA	PAIN
Reference	Fig. 4A,B,C	Fig. 4A	Fig. 4B	Fig. 4C	Fig. 4D
1H freq.	850 MHz	850 MHz	850 MHz	850 MHz	850 MHz
MAS rate	18 kHz	17 kHz	18 kHz	17.5 kHz	17 kHz
t1 max	10 ms	10 ms	10 ms	8 ms	9 ms
t2 max	10 ms	5 ms	12 ms	8 ms	12 ms
1H decoupling	100 kHz	100 kHz	100 kHz	100 kHz	100 kHz
mixing 1	4 ms	3.5 ms	4 ms	4 ms	8 ms

Sample	HET-s(218-289) monomer + [13C,15N] HET-S monomer	HET-s(218-289) monomer + [13C,15N] HET-S monomer	HET-s(218-289) monomer + [13C,15N] HET-S monomer
Spectrum	NCACB	CANCO	NCOCX
1H freq.	850 MHz	850 MHz	850 MHz
MAS rate	17 kHz	18 kHz	18 kHz
t1 max	10 ms	5.5 ms	9.6 ms
t2 max	5 ms	9.6 ms	6.5 ms
t3 max	10 ms	10 ms	10 ms
1H decoupling	100 kHz	100 kHz	100 kHz
mixing 1	3.5 ms CP	3.5 ms CP	4 ms CP
mixing 2	1.2 ms DREAM	4 ms CP	50 ms DARR

[1] Pulse sequences are given in [1]. The DREAM transfer is described in [2].

References

1. Schuetz A, Wasmer C, Habenstein B, Verel R, Greenwald J, et al. (2010) Protocols for the sequential solid-state NMR spectroscopic assignment of a uniformly labeled 25 kDa protein: HET-s(1-227). Chembiochem 11: 1543-1551.
2. Verel R, Ernst M, Meier BH (2001) Adiabatic dipolar recoupling in solid-state NMR: the DREAM scheme. J Magn Reson 150: 81-99.
