# Supplementary material for: The Mechanism of Toxicity in HET-S/HET-s Prion Incompatibility
Source: PLoS Biol. 2012 Dec 27;10(12):e1001451. doi: 10.1371/journal.pbio.1001451 (PMC3531502; doi:10.1371/journal.pbio.1001451)
Supplement: Text S1 — Detailed discussion on the discrepancies between in vivo and in vitro HET-S activity. (PDF) [file pbio.1001451.s007.pdf]

Text S1

Discrepancies between *in vivo* and *in vitro* HET-S activity

Many external factors determine the magnitude of stability the folded state of a protein as well as the reversibility of the unfolding process. Like many proteins, the HeLo domain of HET-S is soluble in the short term at relatively high concentration (30 mg/ml) but it is not stable at any concentration for extended periods, steadily aggregating within days at room temperature. This is a common experience when working with purified proteins due to the irreversibility of many protein-folding processes *in vitro*. *In vivo* there are molecules dedicated to mitigate the detrimental effects of protein aggregation, either through disaggregation activity or by isolating the misfolded proteins in an environment favourable for refolding, thereby avoiding aggregation altogether. HET-S is even less stable than its isolated HeLo domain because the full-length protein can additionally aggregate via its PFD. From these observations we can provide a reasonable explanation for the apparent contradiction: *in vivo*, HET-S cytotoxicity is only achieved upon activation by the HET-s prion because chaperones are continuously working against the process, while *in vitro* or during overexpression in a host that lacks the correct chaperones, HET-S can slowly self-activate via its own PFD or by direct oligomerization of its HeLo domain. Hence, the *in vitro* membrane binding and leakage activity with HET-S in the absence of a prion (although at significantly lower levels) is attributed to the inherent instability of HET-S outside of its natural environment. In *E. coli* we also observed toxicity during expression of just the HeLo domain, HET-S(1-227), which can be due to a high concentration of protein that allows a PFD-independent oligomerization of the HeLo domain. Support for this notion is based on the finding that there is a dimerization interface in the HET-S HeLo domain with a weak dissociation constant (78 µM) [1]. The role of oligomerization via this interface is supported by the fact the HET-S[E86K] variant, whose mutation is at the dimer interface, loses its toxicity and also its ability to dimerize. While the discussed apparent discrepancies can only be rationalized by arguments, it must be noted, that there is a perfect correlation of the phenotype to the *in vitro* activity of the proteins (whether prion-induced or thermodynamically controlled) indicating the discussed activity is biologically relevant.

References

1. Greenwald J, Buhtz C, Ritter C, Kwiatkowski W, Choe S, et al. (2010) The mechanism of prion inhibition by HET-S. Mol Cell 38: 889-899.
